# A Major Histocompatibility Class I Locus Contributes to Multiple Sclerosis Susceptibility Independently from HLA-DRB1*15:01

**DOI:** 10.1371/journal.pone.0011296

**Published:** 2010-06-25

**Authors:** Bruce A. C. Cree, John D. Rioux, Jacob L. McCauley, Pierre-Antoine F. D. Gourraud, Philippe Goyette, Joseph McElroy, Philip De Jager, Adam Santaniello, Timothy J. Vyse, Peter K. Gregersen, Daniel Mirel, David A. Hafler, Jonathan L. Haines, Margaret A. Pericak-Vance, Alastair Compston, Stephen J. Sawcer, Jorge R. Oksenberg, Stephen L. Hauser, IMAGEN, IMSGC

**Affiliations:** 1 Department of Neurology, University of California San Francisco, San Francisco, California, United States of America; 2 Montreal Heart Institute, Montréal, Québec, Canada; 3 Dr. John T. MacDonald Foundation, Department of Human Genetics, Miami University School of Medicine, Miami, Florida, United States of America; 4 Department of Neurology, Brigham and Women's Hospital, Boston, Massachusetts, United States of America; 5 Partners Healthcare Center for Genetics and Genomics, Harvard Medical School, Cambridge, Massachusetts, United States of America; 6 Department of Rheumatology, Hammersmith Hospital, Imperial College London, London, United Kingdom; 7 Robert S. Boas Center for Genomics and Human Genetics, Feinstein Institute for Medical Research, Manhasset, New York, United States of America; 8 Broad Institute of MIT and Harvard, Cambridge, Massachusetts, United States of America; 9 Department of Neurology, Yale University School of Medicine, New Haven, Connecticut, United States of America; 10 Department of Molecular Physiology and Biophysics, Vanderbilt University Medical Center, Nashville, Tennessee, United States of America; 11 Hussman Institute for Human Genomics, Miami University School of Medicine, Miami, Florida, United States of America; 12 Neurology Unit, Department of Clinical Neurosciences, University of Cambridge, Cambridge, United Kingdom; Julius-Maximilians-Universität Würzburg, Germany

## Abstract

**Background:**

In Northern European descended populations, genetic susceptibility for multiple sclerosis (MS) is associated with alleles of the human leukocyte antigen (*HLA*) Class II gene *DRB1*. Whether other major histocompatibility complex (MHC) genes contribute to MS susceptibility is controversial.

**Methodology/Principal Findings:**

A case control analysis was performed using 958 single nucleotide polymorphisms (SNPs) spanning the MHC assayed in two independent datasets. The discovery dataset consisted of 1,018 cases and 1,795 controls and the replication dataset was composed of 1,343 cases and 1,379 controls. The most significantly MS-associated SNP in the discovery dataset was rs3135391, a Class II SNP known to tag the HLA-DRB1*15:01 allele, the primary MS susceptibility allele in the MHC (O.R. = 3.04, *p*<1×10^−78^). To control for the effects of the HLA-DRB1*15:01 haplotype, case control analysis was performed adjusting for this HLA-DRB1*15:01 tagging SNP. After correction for multiple comparisons (false discovery rate = .05) 52 SNPs in the Class I, II and III regions were significantly associated with MS susceptibility in both datasets using the Cochran Armitage trend test. The discovery and replication datasets were merged and subjects carrying the HLA-DRB1*15:01 tagging SNP were excluded. Association tests showed that 48 of the 52 replicated SNPs retained significant associations with MS susceptibility independently of the HLA-DRB1*15:01 as defined by the tagging SNP. 20 Class I SNPs were associated with MS susceptibility with *p*-values ≤1×10^−8^. The most significantly associated SNP was rs4959039, a SNP in the downstream un-translated region of the non-classical *HLA-G* gene (Odds ratio 1.59, 95% CI 1.40, 1.81, *p* = 8.45×10^−13^) and is in linkage disequilibrium with several nearby SNPs. Logistic regression modeling showed that this SNP's contribution to MS susceptibility was independent of the Class II and Class III SNPs identified in this screen.

**Conclusions:**

A MHC Class I locus contributes to MS susceptibility independently of the HLA-DRB1*15:01 haplotype.

## Introduction

The autoimmune disease multiple sclerosis (MS) is one of the leading causes of neurological disability in young adults. Pathologically the disease is characterized by focal areas of inflammation and demyelination (plaques) within the central nervous system with ensuing axonal damage. Although the etiology is not fully understood, MS is a complex genetic disorder and whole genome studies indicate that the major histocompatibility complex (MHC) on chromosome 6p21 represents the strongest genome-wide MS susceptibility locus [1], [2].

In both Northern European and African descended populations, MS susceptibility is associated with alleles of the *HLA* Class II gene *DRB1* [2]–[5] whereas the contribution of other genes within the extended MHC has been controversial [6]–[8]. Extensive linkage disequilibrium (LD) operating in the region [9]–[11], as well as marked polymorphism and high gene density, have complicated efforts to fully resolve the roles of *HLA* and non-*HLA* genes in MS susceptibility. Due to these inherent challenges, a comprehensive approach is needed to refine the contributions of the MHC to genetic risk for MS that includes a large and well-characterized dataset, dense concentration of markers, and appropriate methods to control for the extensive LD across the region.

A panel of single nucleotide polymorphisms (SNPs) selected for moderate LD across the 29 to 34 Mb region of the MHC was employed to map both *HLA* and non-*HLA* disease susceptibility signals [12]. Here we present the results of an analysis of two independent case control MS datasets using 958 SNPs adjusting for the effect of HLA-DRB1*15:01 whose extended haplotype spans the MHC.

## Results

### Case control study

Following quality control, 958 markers were genotyped in both datasets. In the discovery dataset the average number (standard deviation) of missing genotypes for cases was .0040 (.0331) and for controls was .0027 (.0325). In the replication dataset, the average number (standard deviation) of missing genotypes for cases was .0020 (.0060) for controls was .0022 (.0080). There was not a statistically significant difference in missing genotypes between cases and controls in either dataset.

Case control analysis was performed in the discovery dataset composed of 1018 cases and 1795 controls (Table S1) using 958 MHC spanning SNPs (Table S2, see Figure S1 for study design). Population stratification effects were controlled for by including sex and location of subject recruitment (United States versus United Kingdom) in the regression analyses. The Cochran Armitage trend test was used to identify MS associated SNPs and the false discovery rate (FDR = .05) was used to correct for multiple comparisons [13]. The most highly associated SNP was rs3135391 (odds ratio = 3.04, *p*<1×10^−78^), a Class II SNP known to tag the primary MS susceptibility allele HLA-DRB1*15:01 with very high sensitivity and specificity [11].

**Figure 1:**
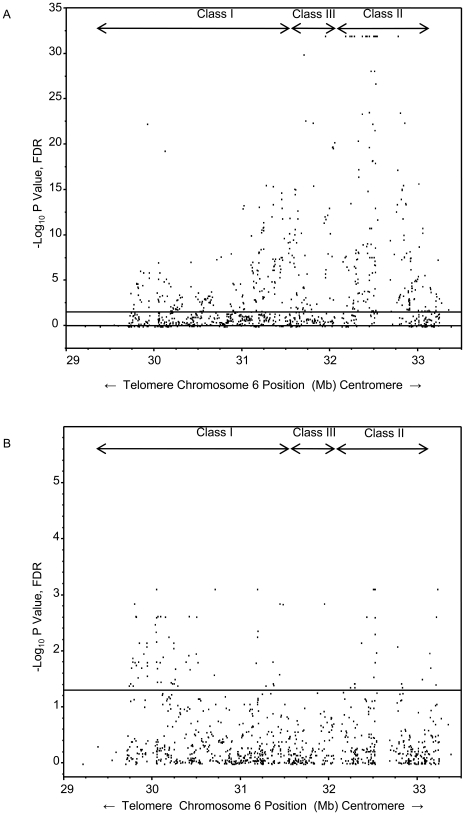
Association test results for 958 SNPs spanning the MHC in the discovery dataset are shown. The location of the SNPs is depicted on the X-axis and the statistical significance of the association is depicted on the Y-axis. A: Discovery dataset (1018 cases and 1795 controls), 958 common SNP subset, FDR = .05, adjusted for sex and center (US versus UK), trend test. B: Discovery dataset, 958 common SNP subset, FDR = .05, adjusted for the HLA-DRB1*15:01 tagging SNP rs3135391), sex and center (US versus UK), trend test.

Using the trend test in the discovery dataset, a total of 501 SNPs in Class I, II and III regions showed statistically significant association with MS susceptibility; most of these associations were likely due to LD within extended haplotypes, particularly the one anchored by the HLA-DRB1*15:01 allele (Figure1A). To correct for the effect of this haplotype, the trend test was performed adjusting for rs3135391 using the 958 SNPs (FDR = .05) and the number of significantly associated SNPs was reduced to 87 (Figure 1B).

A second independent dataset consisting of 1343 cases and 1379 controls was then used to replicate these associations (Table S1). All 958 markers were assessed in the replication dataset with the same association strategy adjusting for the HLA-DRB1*15:01 tagging SNP rs3135391 (FDR = .05). Only markers that were significantly associated in both cohorts, and had the same direction of association, were studied further. 52 such SNPs were significantly associated with MS susceptibility in both datasets (Table S3).

### The merged HLA-DRB1*15:01(-) dataset

A merged cohort was next created by combining the discovery and replication datasets. The MAF for each SNP is reported for cases and controls in the merged dataset as well as the strength of association using the trend test (Table S3). A SNP in the downstream non-coding region of *HLA-G* (rs4959039) was the most significantly associated marker (*p*<8.65×10^−12^) in the merged cohort analysis, after adjusting for the HLA-DRB1*15:01 tagging SNP rs3135391 and potential stratification effects caused by sex, location (US versus UK), and dataset (discovery versus replication).

To further demonstrate that these 52 replicated SNP associations were independent from effects of the extended HLA-DRB1*15:01 haplotype, all subjects carrying at least one copy of this allele, as defined by the tagging SNP rs3135391, were dropped from the merged dataset to create a “HLA-DRB1*15:01(-)” dataset. This excluded a total of 2088 subjects (1277 cases and 811 controls) leaving a HLA-DRB1*15:01(-) dataset that consisted of 1075 cases and 2363 controls. Association tests were performed in this merged HLA-DRB1*15:01(-) dataset and significant associations were found for 48 of the 52 SNPs identified in the case control screens including all previously identified Class I and Class III SNPs (Table S4).

**Figure 2:**
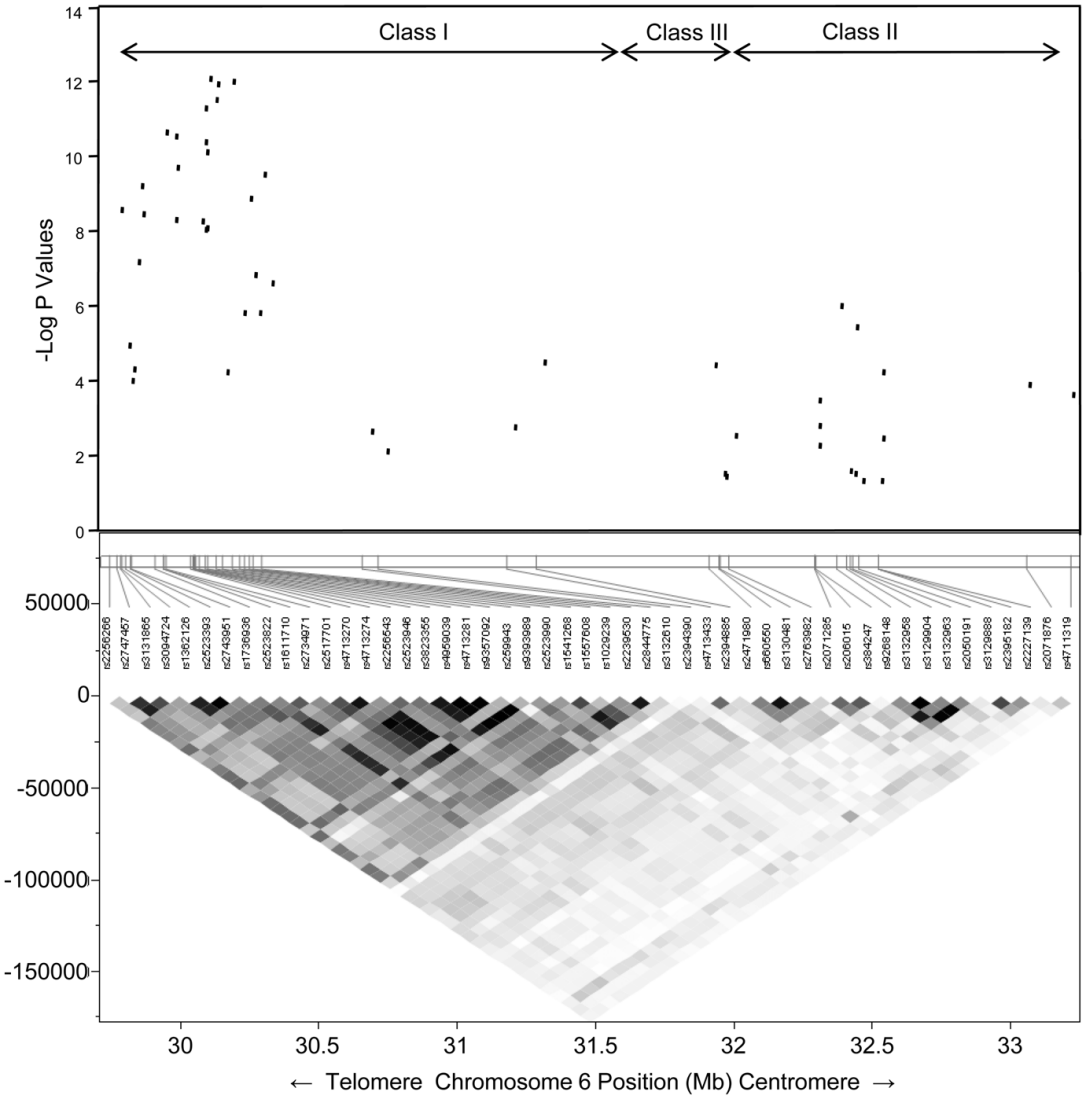
LD map and associations for the 48 SNPs in the merged dataset that excludes all HLA-DRB1*15:01 subjects. Each SNP's position in the MHC is shown on the X-axis with the most telomeric SNPs on the left and the most centromeric SNPs on the right. The lower portion of the figure depicts the strength of LD is in intensity from black to grey to white. Multiple SNPs in the Class I region associated with MS susceptibility independently from HLA-DRB1*15:01 are in moderate to strong LD with each other. These SNPs are in much weaker LD with the MS associated SNPs in the Class III and Class II regions. The degree of statistical significance is depicted in the upper portion of the figure where each SNP's –log_10_ transformed *p*-value is depicted on the Y-axis. The most significant associations with MS susceptibility are in the Class I region and the Class III and Class II signals, although statistically significant, are considerably weaker. An algorithm used to cluster SNPs based on LD-R^2^ [15] grouped together SNPs in the Class III *NOTCH4* gene (rs2071285, rs206015, rs384247) and Class II gene *TSBP* (rs9268148, rs3132958, rs3129904, rs3132963, rs2050191).

Using the genotype test for association in the HLA-DRB1*15:01(-) dataset, 20 Class I SNPs had *p*-values ≤10^−8^ (Table 1). The *HLA-G* linked rs4959039:A>G allele (rs4959039) continued to have the strongest association in this HLA-DRB1*15:01(-) dataset (odds ratio 1.59, 95% confidence intervals 1.40, 1.81, *p*<8.45×10^−13^). Importantly, rs4959039 and the other Class I SNPs associated with MS susceptibility are poorly correlated with the SNPs in the Class III and Class II regions as illustrated by the LD map (Figure 2). For example, the average (range) r^2^ for rs4959039 with the Class III SNPs was .081 (.014 to .149) and for the Class II SNPs was .024 (.002 to .085).

**Table 1:** SNPs associated with MS susceptibility with genome-wide statistical significance in the merged dataset excluding all subjects who carry the HLA-DRB1*15:01 allele listed in order of highest to lowest statistical significance using the Cochran-Armitage trend test for association.

Merged Cohort HLA-DRB1*15:01(-) Subjects								
				MS Associated	Trend	Odds	95% CI	
SNP	Position	Class	Gene	Allele	P Values	Ratio	Lower	Upper
rs4959039	30065047	Class I	HLA-G	A	8.45×10^−13^	1.59	1.40	1.81
rs9393989	30148062	Class I	RNF39	A	9.84×10^−13^	0.63	0.56	0.72
rs9357092	30092230	Class I	HCG9	A	1.17×10^−12^	0.63	0.56	0.72
rs4713281	30086330	Class I	HLA-J	A	3.19×10^−12^	0.63	0.56	0.72
rs4713274	30045471	Class I	MICD	C	5.11×10^−12^	1.56	1.38	1.77
rs1736936	29902295	Class I	HCG4P8	C	2.22×10^−11^	0.70	0.63	0.78
rs2523822	29936638	Class I		A	2.99×10^−11^	1.51	1.34	1.70
rs4713270	30042675	Class I	HCG2P6	A	4.26×10^−11^	0.66	0.58	0.75
rs3823355	30050061	Class I	MICD	C	7.74×10^−11^	1.50	1.33	1.70
rs2734971	29942427	Class I	3.8–1.4	C	2.07×10^−10^	1.42	1.27	1.58
rs2239530	30260093	Class I	TRIM26	C	2.96×10^−10^	0.65	0.57	0.74
rs2523393	29813637	Class I	FLJ35429	C	6.04×10^−10^	0.72	0.65	0.80
rs1541268	30211372	Class I	TRIM40	C	1.40×10^−9^	0.66	0.58	0.76
rs2256266	29740296	Ext Cls I	MOG	A	2.67×10^−9^	0.66	0.58	0.76
rs2743951	29817212	Class I	FLJ35429	C	3.55×10^−9^	1.37	1.24	1.52
rs1611710	29936894	Class I		C	5.06×10^−9^	0.74	0.66	0.82
rs2517701	30033950	Class I	HLA-80	A	5.62×10^−9^	1.41	1.26	1.58
rs2523946	30049921	Class I	MICD	C	8.69×10^−9^	1.36	1.23	1.51
rs2256543	30045811	Class I	MICD	A	9.15×10^−9^	1.36	1.22	1.51
rs1362126	29798997	Class I	HLA-F	A	6.99×10^−9^	0.75	0.67	0.83

In contrast to the poor correlations with the Class III and Class II SNPs, the LD map (Figure 2) shows that some of the associated Class I SNPs are closely linked. SNPs in the Class I region with *p*-values ≤1×10^−8^ that are in moderate to strong LD with each other (as defined by LD-R^2^≥0.5) include: rs2523822, rs2517701 (*HLA-80*), rs4713270 (*HCG2PG*), rs4713274 (*MICD*), rs2523946 (*MICD*), rs3823355 (*MICD*), rs4959039 (in between *HLA-G* and *HLA-A*), rs4713281 (*HLA-J*), rs9357092 (*HCG9*), and rs9393989 (*RNF39*). Using an algorithm to define haplotype blocks by LD-R^2^≥0.5 an apparently separate Class I SNP cluster (rs1362126, rs2523393, rs2743951) emerges that includes a tagging SNP for the HLA-B*44:02 allele (rs2523393), a recently identified MS protective allele [12], [14].

### Tests for independent association using logistic regression models

To confirm that the contribution to MS susceptibility of the rs4959039 SNP was independent of any residual Class II associations, logistic regression models were constructed. Because many of the 48 SNPs associated with MS susceptibility in the HLA-DRB1*15:01(-) dataset are in moderate to strong LD with each other a clustering algorithm was used to group the 48 SNPs into 20 clusters (LD-R^2^≥.05) and identify SNPs that tagged each cluster (Table S5) [15].

Logistic regression with backwards stepwise selection was then used with the 20 tagged SNPs and covariates to control for population stratification, i.e. sex and dataset (discovery versus replication). Using the trend model, the rs4959039 SNP was significantly associated with MS susceptibility (*p* = 3.70×10^−10^, odds ratio  = 1.54), despite controlling for the cumulative effects of Class II SNPs. Further logistic regression modeling showed that the rs4959039 MS association was also independent of the Class III associated SNPs. When the Class II and Class III SNPs were included in the logistic regression model, the rs4959039 SNP retained a highly significant association with MS susceptibility (*p* = 9.70×10^−10^, odds ratio  = 1.52).

To estimate the contributions of the 20 Class I, II and III SNP clusters to MS susceptibility a model was constructed entering all 20 SNPs, plus covariates to control for stratification effects. Backwards stepwise selection was used to refine the model so that only variables with *p*-values ≤.01 were retained in the model. In the final model, SNP rs4959039 maintained the most statistically significant contribution (*p*<4.80×10^−10^, odds ratio = 1.53). Three Class II SNPs rs3132963 (*p*<1.59×10^−5^, odds ratio = 1.65), rs2227139, (*p*<.00135, odds ratio = 1.20) and rs4711319 (*p*<.00125, odds ratio = 1.28) were retained in the model suggesting residual independent Class II contributions. The area under the receiver operator curve for this model was .634 whereas the area under the receiver operator curve modeling the rs4959039 SNP alone was .617 showing that the contribution of these Class II SNPs is modest. Importantly, during the backward stepwise selection process all other Class I SNP clusters were dropped from the model suggesting that the Class I contribution to MS susceptibility is driven by the SNP cluster tagged by rs4959039.

Logistic regression was used to determine whether the association of the rs4959039 SNP was dependent on the rs2523393 SNP (tags HLA-B*44:02). Despite their close physical proximity, the association of the rs4959039 SNP remained highly significant (*p* = 6.10×10^−6^, odds ratio  = 1.43) after adjusting for the effect of the rs2523393 SNP whereas the association of the rs2523393 SNP (that tags the MS protective allele HLA-B*44:02) was attenuated (*p* = .015, odds ratio  = .85).

### Two-locus Class I haplotypes

To further understand the contributions of these loci to MS susceptibility two-locus haplotypes were constructed for SNPs rs2523393 (the HLA-B*44:02 tagging SNP) and rs4959039 (Table S6). This analysis defined a MS risk haplotype as rs2523393:T>C with rs45959039:A>G and the converse MS protective haplotype as rs2523393:C>T with rs45959039:G>A. Due to LD this analysis could not definitively prove that the influence of these loci on MS risk was independent. However, the heterozygous haplotype appears to be protective for MS risk (odds ratio = .71, *p*<9.73×10^−5^) indicating that the protective haplotype is dominant.

### Transmission disequilibrium test in HLA-DRB1*15:01(-) trio families

As an additional test of association, the rs4959039 was assessed using the transmission disequilibrium test in a subset of the discovery dataset for whom parental genotyping was available. 347 trio families (affected individual plus both parents) that did not carry the HLA-DRB1*15:01 allele were genotyped for the rs4959039 SNP. The chromosome carrying the allele of rs4959039:A>G was transmitted 112 times and not transmitted 81 times in heterozygous trio families. Despite the small size of this family based dataset, a borderline level of statistical significance was observed (*p* = .046) supporting the validity of this SNP as an MS susceptibility locus using a family-based association test.

To determine whether the rs4959039:A>G allele adds to the risk of MS in HLA-DRB1*15:01 subjects, bi-allelic haplotypes for rs3135391:T>C (the SNP that tags HLA-DRB1*15:01) and rs4959039:A>G individuals were constructed in the merged dataset (Table 2). Each bi-allelic haplotype was treated as a dichotomous variable in this analysis. The presence of the rs4959039:A>G allele contributed to MS susceptibility both in subjects who carry the HLA-DRB1*15:01 allele as well as those that do not. In addition, the rs4959039:A>G allele appears to be additive to the effect of HLA-DRB1*15:01 increasing the odds ratio for MS from 5.89 to 6.46, although the confidence intervals for the odds ratios of these haplotypes overlap.

**Table 2:** Paired marker analysis for HLA-DRB1*15:01 and rs4959039 haplotypes in the merged dataset.

*HLA-DRB1*	rs4959039	N	O.R.	95 C.I. lower	95 C.I. upper	*p*-Value
HLA-DRB1*X	“G”	3226				
HLA-DRB1*15:01	“G”	129	5.89	3.62	9.59	1.036×10^−12^
HLA-DRB1*15:01	“A”	1954	6.46	4.57	9.13	4.95×10^−26^
HLA-DRB1*X	“A”	216	1.90	1.35	2.67	.0002

Two locus haplotypes were constructed and the odds ratio for association with MS susceptibility for each haplotype was tested in a logistic regression model treating each haplotype as a categorical variable. The odds ratio for the HLA-DRB1*15:01 allele in the merged dataset was 3.50 (*p*<1.46×10^−100^). All results are adjusted for stratification effects caused by sex, location (US versus UK) and dataset (discovery versus replication). DRB1*X refers to subjects who do not carry the HLA-DRB1*15:01 allele.

### *HLA-G* SNP associations from a meta-analysis genome-wide association study

Depending on the reference sequence the SNP rs4959039 maps to non-coding regions centromeric to *HLA-G* or *HLA-A*. The chromosome 6 cox reference sequence places this SNP in the intergenic non-coding region centromeric to *HLA-G* whereas the chromosome 6 qb1 reference sequence maps the SNP centromeric to *HLA-A*. It appears that this SNP tags a possible ancestral duplication near both genes [16]. This observation raises the question as to whether the MS susceptibility signal associated with this SNP arises from alleles of *HLA-G*, *HLA-A*, or other nearby genes. Indeed, as presented above, many of the Class I SNPs identified in this study are in moderate to strong LD with each other.

A panel of different SNPs in the *HLA-G* locus was assessed using a dataset described in a recent genome wide association scan (GWAS) meta-analysis [14]. Although the published GWAS meta-analysis included subjects from the discovery dataset, these subjects were excluded from the following analysis to create an independent dataset consisting of 1606 MS cases and 5425 controls. In the GWAS meta-analysis 167 SNPs mapped to the *HLA-G* locus. After adjusting for HLA-DRB1*15:01 using a tagging SNP and sex 63 of the 167 SNPs were associated with MS susceptibility with *p-*values ≤.01 (Table S7). The majority of the SNPs mapped to the untranslated region centromeric to *HLA-G*, some with *p*-Values ≤1×10^−6^ (rs1611715, rs3115627, rs2734982, rs2975033). 6 SNPs map within the *HLA-G* gene itself with *p*-Values ≤1×10^−4^. SNPs rs1611627, rs915668, rs 1736920 and rs1632933 are intronic SNPs whereas SNP rs1063320 maps to the 3′ end of the last exon of *HLA-G* and is transcribed but not translated. These data are consistent with the proposition that a MHC Class I MS susceptibility locus that is independent of the extended HLA-DRB1*15:01 haplotype maps to the region of the *HLA-G* gene.

### Summary

This comprehensive SNP based analysis spanning the 29 to 34 kb region of the MHC shows that 52 SNPs in Class I, II and III regions of the MHC were associated with MS susceptibility in two independent datasets. Moreover, 20 of these SNPs were associated with MS susceptibility with *p*-values <1×10^−8^ in a dataset that does not carry the extended HLA-DRB1*15 haplotype. The most significant association was with rs4959039, a class I SNP near *HLA-G*. The association of this SNP with MS susceptibility appears to be independent of the effects of the other identified Class II and Class III SNPs.

## Discussion

Using two case control datasets and a panel of SNPs specifically selected to capture the genetic variation within the MHC region we found that the MHC locus contributes to MS susceptibility, not only through the well recognized effect of HLA-DRB1*15:01, but also through independent contributions from a Class I locus. This study proves that, after the HLA-DRB1*15:01 extended haplotype, the Class I region is the most significant contributor to MS susceptibility within the MHC. Importantly, these observations contrast with an earlier publication of a Canadian cohort which concluded that all Class I associations with MS susceptibility were due to LD with HLA-DRB1*15:01 [5]. Although genetic heterogeneity might account for these differences, it is more likely that the structure of the current study, specifically the large dataset and denser set of informative markers, made possible the detection of independent effects of Class I and Class III genes.

### Class I genes and MS susceptibility

The strongest HLA-DRB1*15:01 independent MS association was with rs4959039, a SNP near the non-classical *HLA-G* gene. Several other SNPs in neighboring pseudogenes *HLA-80*, *HCG2P6*, *MICD* and *HLA-J* were also associated with MS susceptibility and are in LD with the rs4959039 SNP. These SNPs are not strongly linked to the SNP that tags the recently identified Class I MS protective allele HLA-B*44:02 [12], [14] and are independent of the major MS susceptibility allele HLA-DRB1*15:01. Because of the prohibitive cost we were unable to genotype classical *HLA* alleles in these large datasets to control for the possible contributions of HLA-DRB1*0301 [17] or other *HLA-DRB1* alleles. Nevertheless, logistic regression models that controlled for the 10 most statistically significant Class II SNPs, as well as the 8 Class III SNPs identified in this study, demonstrated an independent allelic contribution of rs4959039 to MS susceptibility.

Although this association study cannot exclude the possibility that another closely linked MHC Class I gene, or genes, gives rise to the MS susceptibility signal detected by the rs4959039 SNP it is clearly of interest that this SNP is in the 3′ un-translated region of *HLA-G*. We conclusively demonstrated that this SNP's association with MS susceptibility is independent of HLA-DRB1*15:01 and provided evidence that this SNP is not tightly linked to any of the Class III or Class II associations identified in this screen.

However, the rs4959039 SNP also maps to a duplication that is near *HLA-A*. *HLA-A* alleles were previously associated with MS susceptibility: the HLA*03 allele is thought to increase MS risk in HLA-DRB1*15:01 subjects [18], [19] whereas the HLA-A*02 allele is thought to reduce MS risk [20]. Several lines of evidence suggest that the rs4959039 SNP's association with MS might be through *HLA-G* rather than HLA-A*03. First, the HLA-A*03 allele is part of the extended HLA-DRB1*15:01 haplotype that was effectively excluded in this study. Second, the HLA-A*03 allele that was imputed in the discovery dataset is not tightly correlated with the rs4959039 SNP (r^2^ = .002). Third, SNPs in *HLA-A* were not identified as disease-associated in either the discovery or the replication datasets. Lastly, using a different panel of *HLA-G* imputed SNPs from a genome wide meta-analysis in an independent dataset, multiple SNPs in the *HLA-G* locus were significantly associated with MS susceptibility after adjusting for HLA-DRB1*15:01. Thus we interpret our results as suggesting that the rs4959039 SNP association with MS risk is not through HLA-A*03. However, because typing of class I genes was unavailable for nearly the entire dataset we were unable to further analyze the relationship between the rs4959039 SNP and HLA-A*02, or other *HLA-A* alleles. Given that SNP rs4959039 tags a large haplotype block that includes *HLA-A*, mapping the class I susceptibility gene, or genes, will not only require classical typing of *HLA-A* but also could require an even larger dataset that excludes HLA-DRB1*15:01 carriers. For this reason, functional studies of *HLA-A* and *HLA-G* associated variants in MS patients will likely be useful to understand how alleles of these genes influence MS risk.

*HLA-G* is a biologically interesting candidate gene because of its prominent function in immune tolerance. HLA-G is a non-classical, HLA Class I molecule characterized by relatively limited polymorphism and alternate splice sites that result in several membrane bound and soluble isoforms [21]. The *HLA-G* gene includes 42 alleles at the DNA level, 14 alleles at the protein level, and 2 null alleles based on sequence variation in exons 2–4 (the _1 to _3 domains) [22]. In theory, polymorphisms affecting the *HLA-G* primary sequence, differences in alternate splicing and expression pattern, could promote or reduce immune tolerance and in this manner influence MS susceptibility. Prior genetic studies of *HLA-G* in MS susceptibility found conflicting results. One study found no association of three *HLA-G* alleles and MS susceptibility [23] whereas another found an association of an *HLA-G* promoter polymorphism with MS susceptibility by the transmission distortion test [8]. Both studies were limited by relatively small sample sizes and few genetic markers.

In contrast to the ubiquitous expression of HLA-A, HLA-B and HLA-C, HLA-G is found primarily in extravillous trophoblasts: fetal cells that invade the maternal decidua during placenta formation [24], [25]. These fetal trophoblasts are thought to play a role in inducing maternal tolerance for the fetus. HLA-G probably does not function in antigen presentation to HLA class restricted T cells [26]. Rather, HLA-G binds to and stimulates signaling via the leukocyte immunoglobulin-like receptors (LILRB1/ILT2/CD85j) as well as LILRB2/ILT4/CD85d) and KIR2DL4 (CD158d) [25]. These cell surface receptors are expressed on antigen presenting cells such as dendritic cells, macrophages and B cells and are also found on natural killer (NK) cells, T cells, eosinophils, and osteoclasts. Although not well understood, LILRB signaling inhibits co-stimulation of T cell responses during antigen presentation [27]. When expressed on target cells HLA-G inhibits NK cell killing of the target cell by stimulation of inhibitory pathways [28]. These observations suggest that HLA-G has an important role in inducing maternal-fetal tolerance. Additional support for role of HLA-G in immune tolerance comes from murine allogenic tissue graft experiments in which HLA-G expression prolongs graft survival [29].

Whether HLA-G is involved in induction of immune tolerance in other body tissues, or disease states, is somewhat controversial. Some authors challenge the idea that HLA-G is expressed anywhere other than the trophobast [30]. However, a growing body of evidence suggests that HLA-G has an important role in preventing immunological targeting of malignant cells [31]. Furthermore, HLA-G may have important roles in inflammatory skin conditions [32] and myopathies [33].

A role for HLA-G in multiple sclerosis pathogenesis was first proposed based on the observation that sHLA-G levels were elevated in MS patients relative to healthy controls [34]. Furthermore, sHLA-G is down-regulated in patients who have actively inflamed MS plaques as evidenced by gadolinium-DPTA enhancement on brain MRI imaging [35]. HLA-G is known to be strongly expressed in brain specimens from MS patients where it is present in acute inflammatory demyelinating plaques, chronic active plaques, peri-plaque areas and normal appearing white matter [36]. In MS, HLA-G is expressed primarily on microglia, macrophages, and endothelial cells. In addition to HLA-G, one of its receptors, LILRB1/ILT2, is also found in MS brain tissue suggesting that HLA-G expression in MS brain is functionally relevant, possibly through an inhibitory feedback pathway directed at down regulating pro-inflammatory T cells. Recently, HLA-G^pos^ T_reg_ cells were identified in MS cerebrospinal fluid, as well as in inflammatory brain tissue, and these cells are thought to function as suppressor cells, counterbalancing the tissue destructive effects of autoimmune inflammation [37]. Taken together, these observations suggest that HLA-G may have a fundamental role in limiting tissue injury in MS by regulating auto-reactive immune cells within the central nervous system.

Thus, it is possible that a *HLA-G* associated haplotype could contribute to MS risk by influencing signaling via LILRB1/ILT2 or the KIR2DL4 natural killer (NK) receptors [38]. Polymorphisms in *HLA-G* or *KIR2DL4* could influence CD56^bright^ NK cell function whose corresponding immunoregulatory pathway involves the already established MS susceptibility genes, the interleukin 2 receptor (*IL2RA*) and interleukin 7 receptor (*IL7R*) [2].

### Other Class I loci

In addition to the rs4959039 (near *HLA-G*) association, several other Class I SNPs associated with MS susceptibility were identified, replicated and shown to have HLA-DRB1*15:01 independent effects. One group of SNPs tags the HLA-B*44:02 allele. Tagging SNPs for the closely linked HLA-C*0501 allele [7] did not survive the stringent criteria for association used in this study. These SNPs narrowly missed the cutoff for inclusion as candidates in the discovery dataset screen but were associated with MS susceptibility in the replication dataset screen. When these SNPs were included in the merged HLA-DRB1*15:01(-) dataset, tagging SNPs for HLA-C*05:01 [7] were significantly associated with MS susceptibility (data not shown). Logistic regression modeling suggested that the primary signal in the Class I region arises from the locus identified by SNP rs4595039 although it remains possible that there could be independent contributions from other Class I loci.

Both HLA-C*05:01 and HLA-B*44:02 are reportedly protective alleles for MS susceptibility [7], [12], [14]. These neighboring alleles are in tight LD making discrimination between the effects of each allele challenging. In addition, different alleles of HLA-A may influence MS susceptibility in opposite directions. HLA-A*0301 may in crease MS risk; however, this allele is part of the expanded haplotype shared by HLA-DRB1*15:01 and its proposed influence on MS susceptibility may be confounded by linkage to HLA-DRB1*15:01 [6]. In contrast, HLA-A*02:01 appears to have a protective effect [20]. This allele is also linked to the SNP identified in the present study, rs4595039. Functional studies, or fine mapping studies in populations with different patterns of LD, will be needed to determine whether the protective effect proposed for HLA-A*02:01 is mediated by linkage to an allele of *HLA-G* or other neighboring genes.

In summary, we found MHC SNP associations with MS susceptibility, independent from the primary influence of HLA-DRB1*15:01, in the Class I, Class II and Class III regions. The most significant contribution arises from the Class I region in the vicinity of the *HLA-G* gene. *HLA-G*, or another closely linked gene such as *HLA-A*, contributes to MS risk independently from the recently identified Class I allele HLA-B*44:02, as well as other Class II and Class III SNPs identified in the present study. Thus a Class I locus near *HLA-G/HLA-A* is a replicated locus within the MHC that contributes to MS risk independently of HLA-DRB1*15:01. The possible *HLA-G* association is particularly interesting because HLA-G is thought to function in induction of immune tolerance and is highly expressed in MS brain tissue. Further studies of functional polymorphisms in *HLA-G*, classical HLA typing, as well as studies in populations with different patterns of LD within the MHC, will help further define this locus's contribution to MS risk.

## Methods

All study subjects signed written informed consent forms approved by the following local institutional review boards in accordance with the Declaration of Helsinki: Committee on Human Research (UCSF), CERDNT (MHI), Human Subjects Research Office (University of Miami), Partners Healthcare IB/Human Research Office, North Thames MREC, The North Shore - LIJ Health System IRB, Vanderbilt HRPP and Berkshire Research Ethics Committee.

The MS discovery dataset consists of 1018 cases (520 from the US and 498 from the UK) and 1795 controls (1049 from the US and 746 from the UK). All MS subjects met International Panel criteria for multiple sclerosis [39]. The control population was composed of samples from the United Kingdom 1958 birth cohort as well as a cohort of healthy subjects form The New York Cancer Project. The family based trio analysis was conducted on a subset of 347 trio families (MS patient and both parents) from the discovery cohort who did not carry the HLA-DRB1*15:01 tagging SNP.

The genetic marker analysis used for the discovery cohort was a custom Illumina array that composed of 1337 SNPS to tag common SNP variation across the 3.44 Mb of the MHC. These SNPs were selected using the Tagger algorithm for having relatively low LD from approximately 7000 SNPs spanning the MHC [11], [40]. Overall this set of SNPs captured variation of common (≥5%) HLA markers, less-common (<5%) HLA markers, common non-HLA markers, and less-common non-HLA markers, with an average maximum *r^2^* of 0.80, 0.64, 0.90, and 0.62, respectively. [14] The genetic marker analysis used for the replication cohort was a custom Illumina array that included the 1337 SNPS used to tag common SNP variation across the 3.44 MB of the MHC, 29 to 44 Mb as well as other SNPs in genes of interest that are neither described nor analyzed in the present manuscript. The HLA-DRB1*15:01 (negative) dataset had .98 power (α = .05) to detect the association of the rs4959039 SNP with the class I MS susceptibility locus, assuming that this SNP was tightly linked to the locus with D' = .8 and using the odds ratio and minor allele frequencies associated with this SNP in this dataset.

A multi-step quality control (QC) strategy was employed for the samples and SNPs using the following strategy for both discovery and replication cohorts.

1. Samples whose call rate was <75% were removed
2. SNPs whose call rate was <60% in each group were removed
3. Samples in which there was evidence of contamination as estimated by π≥0.1 using IBD/IBS statistics were removed
4. SNPs with minor allele frequency (MAF) <1% were removed
5. SNPs where HWE <0.01 in the datasets (cases and controls) were flagged
6. Only SNPs that passed QC in both the discovery and replication datasets were included

Following the QC strategy, 16 MS cases were removed, yielding a total of 1018 cases available for the discovery case control study. The replication dataset consisted of an additional 1343 cases and 1379 controls from the US and UK. Of the 1337 Illumina SNPs, 958 passed QC in both datasets.

### Marker Trait Association

Associations with MS susceptibility with SNPs and imputed alleles were assessed by the Cochran Armitage trend tests using the false discovery rate method to control for multiple comparisons [13]. SAS, JMP® genomics (Cary, NC) and STATA 9 (North Fork, TX) were used to perform statistical analyses. Population stratification caused by differences in markers between the sexes, the country of the subject's origin (United States versus United Kingdom) and dataset (discovery versus replication) was controlled for by inclusion of these covariates as fixed effects in the regression analyses.

Following identification of SNPs that were significantly associated with MS susceptibility in both datasets the discovery and replication datasets were merged and subjects carrying the rs3135391:T>C SNP that tags the HLA-DRB1*15:01 allele were excluded. MS associated SNPs where (Hardy Weinberg Equilibrium) HWE <0.01 in the control population of the merged dataset were dropped. 52 SNPs were significantly associated with MS susceptibility in both datasets.

In the discovery dataset previous 2- or 4-digit typing of *HLA-DRB1* was available for 27.6% of the dataset (N = 777). [14] In this subset, the tagging SNP rs3135391 was 100% sensitive and 100% specific for correctly calling HLA-DRB1*15:01. HLA typing was performed by different methodologies, including PCR-based sequence-specific oligonucleotide probe reverse-line blot assay, sequence-specific oligonucleotide (LABType) typing, and exons 2/3 sequence based typing.

## Supporting Information

Figure S1 Study design summary. The 958 SNPs spanning the MHC used in the initial screens are listed in Supplemental Table 2. The 48 SNPs associated with MS in both datasets are listed in Supplemental Table 3 and the 48 SNPs with *p*-values ≤1×10^−8^ in the merged HLA-DRB1*15:01(-) dataset are listed in Table 1.
(0.10 MB TIF)

Table S1 Table S1. Case control datasets: The proportion of women to men in the control populations was well matched at the two study centers. However, the proportion of women to men in the MS subjects was significantly increased in the UK dataset.
(0.07 MB DOC)

Table S2 Table S2: 958 SNPs genotyped in both discovery and replication datasets. Ext = extended.
(0.17 MB DOC)

Table S3 Table S3: 52 SNPs significantly associated with MS susceptibility in the discovery and replication datasets using Cochran Armitage trend test, FDR = .05, adjusted for sex, center (US versus UK) and HLA-DRB1*15:01. The SNPs are listed in order of chromosomal position from telomere to centromere. The *p*-values for the merged dataset are unadjusted. rs2523393 is a tagging SNP for HLA-B*44:02 [12], [14].
(0.17 MB DOC)

Table S4 Table S4: 48 SNPs significantly associated with MS susceptibility in the merged HLA-DRB1*15:01 (-) dataset, using the trend test and adjusting for sex, center (US versus UK) and dataset (discovery versus replication). SNPs are listed in order of most to least statistical significance. Four class II SNPs identified in the discovery and replication datasets were no longer significantly associated with MS susceptibility in the HLA-DRB1*15:01 (-) dataset: rs3129961, rs3135352, rs3135391, and rs3135388.
(0.14 MB DOC)

Table S5 Table S5: 48 SNPs that are associated with MS susceptibility in the HLA-DRB1*15:01(-) dataset are grouped together using an algorithm to define SNP clusters based on LD-R^2^≥.05 (moderate to strong LD) [15]. The 48 SNPs can be grouped into 20 SNP clusters and tagging SNPs for each cluster are designated by an asterisk. The SNPs are listed in order of cluster size with the largest cluster including 10 SNPs and the smallest SNP clusters include only single SNPs.
(0.09 MB DOC)

Table S6 Table S6: Two locus haplotypes for the SNPs rs2523393 (tags HLA-B*44:02) and SNP rs459039 (near *HLA-G*). A MS risk haplotype is rs2523393:T>C with rs4959039:A>G and a MS protective haplotype is rs2523393:C>T with rs4959039:G>A. The heterozygous haplotype is appears to be protective suggesting a dominant effect of the protective haplotype. The *p*-values and odds ratios are adjusted for the covariates sex (men versus women) and cohort (discovery versus replication) to control for stratification.
(0.05 MB DOC)

Table S7 Table S7: SNPs significantly associated with MS susceptibility in the *HLA-G* locus typed in an independent dataset used for a genome wide meta-analysis.
(0.12 MB DOC)
