# Supplementary figures and images for: A Major Histocompatibility Class I Locus Contributes to Multiple Sclerosis Susceptibility Independently from HLA-DRB1*15:01
Source: PLoS One. 2010 Jun 25;5(6):e11296. doi: 10.1371/journal.pone.0011296 (PMC2892470; doi:10.1371/journal.pone.0011296)

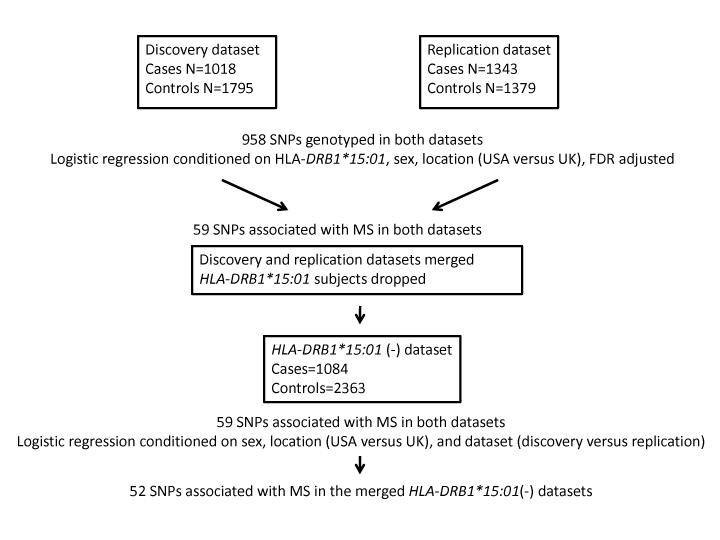

Supplement: Figure S1 — Study design summary. The 958 SNPs spanning the MHC used in the initial screens are listed in Supplemental Table 2. The 48 SNPs associated with MS in both datasets are listed in Supplemental Table 3 and the 48 SNPs with p-values ≤1×10^−8^ in the merged HLA-DRB1*15:01(-) dataset are listed in Table 1. (0.10 MB TIF) [file pone.0011296.s001.tif]
