# Supplementary material for: A Major Histocompatibility Class I Locus Contributes to Multiple Sclerosis Susceptibility Independently from HLA-DRB1*15:01
Source: PLoS One. 2010 Jun 25;5(6):e11296. doi: 10.1371/journal.pone.0011296 (PMC2892470; doi:10.1371/journal.pone.0011296)
Supplement: Table S1 — Case control datasets: The proportion of women to men in the control populations was well matched at the two study centers. However, the proportion of women to men in the MS subjects was significantly increased in the UK dataset. (0.07 MB DOC) [file pone.0011296.s002.doc]

| Discovery dataset | | | |
| --- | --- | --- | --- |
| US | Case | 520 | Women: 353 (67.9%)  Men: 167 (32.1%) |
|  | Control | 1049 | Women: 784 (74.7%)  Men: 265 (25.3%) |
|  |  |  |  |
| UK | Case | 498 | Women: 377 (75.7%)  Men: 121 (24.3%) |
|  | Control | 746 | Women: 571 (76.5%)  Men: 175 (23.5%) |

| Replication dataset | | | |
| --- | --- | --- | --- |
| US | Case | 669 | Women: 344 (51.4%)  Men: 325 (48.6%) |
|  | Control | 615 | Women: 329 (53.5%)  Men: 286 (46.5%) |
|  |  |  |  |
| UK | Case | 674 | Women: 493 (73.1%)  Men: 181 (26.9%) |
|  | Control | 764 | Women: 568 (74.3%)  Men: 196 (25.7%) |

| Merged *HLA-DRB1(-)* dataset | | | |
| --- | --- | --- | --- |
| Discovery | Case | 448 | Women: 306 (68.3%)  Men: 142 (31.7%) |
|  | Control | 1333 | Women: 1,002 (75.2%)  Men: 331 (24.8%) |
|  |  |  |  |
| Replication | Case | 636 | Women: 379 (59.6%)  Men: 257 (40.4%) |
|  | Control | 1030 | Women: 660 (64.1%)  Men: 370 (35.9%) |
