# Supplementary material for: A Major Histocompatibility Class I Locus Contributes to Multiple Sclerosis Susceptibility Independently from HLA-DRB1*15:01
Source: PLoS One. 2010 Jun 25;5(6):e11296. doi: 10.1371/journal.pone.0011296 (PMC2892470; doi:10.1371/journal.pone.0011296)
Supplement: Table S2 — 958 SNPs genotyped in both discovery and replication datasets. Ext = extended. (0.17 MB DOC) [file pone.0011296.s003.doc]

| **SNP** | **Position** | **Class** | **Gene** | **Function** |
| --- | --- | --- | --- | --- |
| rs9295794 | 29212913 | Ext Class I | OR2N1P | olfactory receptor, family 2, subfamily N, member 1 pseudogene |
| rs9257694 | 29382464 | Ext Class I | OR5U1 | olfactory receptor, family 5, subfamily U, member 1 |
| rs2746149 | 29543333 | Ext Class I | OR2H1 | olfactory receptor, family 2, subfamily H, member 1 |
| rs3094572 | 29588971 | Ext Class I |  |  |
| rs2267635 | 29700409 | Ext Class I | GABBR1 | gamma-aminobutyric acid (GABA) B receptor, 1 |
| rs29243 | 29707080 | Ext Class I | GABBR1 | gamma-aminobutyric acid (GABA) B receptor, 1 |
| rs3025623 | 29715725 | Ext Class I | SUMO2P | SMT3 suppressor of mif 2, 3 homolog 2 (S. cerevisiae) pseudogene |
| rs1233374 | 29716672 | Ext Class I |  |  |
| rs29233 | 29719207 | Ext Class I |  |  |
| rs29232 | 29719409 | Ext Class I |  |  |
| rs3129073 | 29723800 | Ext Class I |  |  |
| rs29269 | 29725725 | Ext Class I |  |  |
| rs29272 | 29726344 | Ext Class I |  |  |
| rs29234 | 29732090 | Ext Class I | MOG | myelin oligodendrocyte glycoprotein |
| rs3130250 | 29732979 | Ext Class I | MOG | myelin oligodendrocyte glycoprotein |
| rs3117286 | 29737752 | Ext Class I | MOG | myelin oligodendrocyte glycoprotein |
| rs2256266 | 29740296 | Ext Class I | MOG | myelin oligodendrocyte glycoprotein |
| rs2071652 | 29743295 | Ext Class I | MOG | myelin oligodendrocyte glycoprotein |
| rs2535238 | 29753016 | Ext Class I | ZFP57 | zinc finger protein 57 homolog (mouse) |
| rs445150 | 29754857 | Ext Class I | ZFP57 | zinc finger protein 57 homolog (mouse) |
| rs2535236 | 29759663 | class I | ZFP57 | zinc finger protein 57 homolog (mouse) |
| rs2747454 | 29763091 | class I |  |  |
| rs2747457 | 29764395 | class I |  |  |
| rs3129029 | 29770421 | class I |  |  |
| rs7776082 | 29775251 | class I |  |  |
| rs2107203 | 29777808 | class I |  |  |
| rs3131865 | 29780143 | class I |  |  |
| rs3094727 | 29780507 | class I |  |  |
| rs3094724 | 29782273 | class I |  |  |
| rs1632962 | 29791786 | class I |  |  |
| rs1362126 | 29798997 | class I | HLA-F | MHC, class I, F |
| rs1736926 | 29800540 | class I | HLA-F | MHC, class I, F |
| rs1628578 | 29803480 | class I | RPL23AP1 | ribosomal protein L23a pseudogene 1 |
| rs9258208 | 29812523 | class I | FLJ35429 | hypothetical protein FLJ35429 |
| rs7751815 | 29813062 | class I | FLJ35429 | hypothetical protein FLJ35429 |
| rs2523393 | 29813637 | class I | FLJ35429 | hypothetical protein FLJ35429 |
| rs932338 | 29814911 | class I | FLJ35429 | hypothetical protein FLJ35429 |
| rs2743951 | 29817212 | class I | FLJ35429 | hypothetical protein FLJ35429 |
| rs730858 | 29818606 | class I | FLJ35429 | hypothetical protein FLJ35429 |
| rs2072894 | 29821014 | class I | FLJ35429 | hypothetical protein FLJ35429 |
| rs1633086 | 29823740 | class I | FLJ35429 | hypothetical protein FLJ35429 |
| rs1633085 | 29823975 | class I | FLJ35429 | hypothetical protein FLJ35429 |
| rs3823318 | 29826556 | class I | IFITM4P | interferon induced transmembrane protein 4 pseudogene |
| rs4713243 | 29828939 | class I |  |  |
| rs422343 | 29840408 | class I |  |  |
| rs1737046 | 29843671 | class I |  |  |
| rs1362070 | 29850277 | class I | HCP5P14 | HLA complex P5 pseudogene 14 |
| rs1737008 | 29866095 | class I | HCP5P13 | HLA complex P5 pseudogene 13 |
| rs1633005 | 29872450 | class I | HCP5P13 | HLA complex P5 pseudogene 13 |
| rs1736981 | 29878300 | class I | RPL7AP7 | ribosomal protein L7a pseudogene 7 |
| rs1736976 | 29881977 | class I | RPL7AP7 | ribosomal protein L7a pseudogene 7 |
| rs2523408 | 29886087 | class I | MICG | MHC class I polypeptide-related sequence G pseudogene |
| rs1736959 | 29890448 | class I | MICG | MHC class I polypeptide-related sequence G pseudogene |
| rs1736936 | 29902295 | class I | HCG4P8 | HLA complex group 4 pseudogene 8 |
| rs1736935 | 29902421 | class I | HCG4P8 | HLA complex group 4 pseudogene 8 |
| rs2735014 | 29913787 | class I | HLA-G | HLA-G histocompatibility antigen, class I, G |
| rs2735005 | 29916442 | class I |  |  |
| rs1611133 | 29917360 | class I |  |  |
| rs2394186 | 29924399 | class I |  |  |
| rs2734984 | 29928809 | class I | MICF | MHC class I polypeptide-related sequence F pseudogene |
| rs3094657 | 29936288 | class I |  |  |
| rs2523822 | 29936638 | class I |  |  |
| rs1611710 | 29936894 | class I |  |  |
| rs3094165 | 29941519 | class I |  |  |
| rs2734971 | 29942427 | class I | 3.8-1.4 | 3.8-1.4 pseudogene |
| rs3094161 | 29943051 | class I |  |  |
| rs886398 | 29947246 | class I | HCP5P10 | HLA complex P5 pseudogene 10 |
| rs2524005 | 30007655 | class I | HLA-K | MHC, class I, K |
| rs3893538 | 30032403 | class I | HLA-80 | HLA-80 pseudogene |
| rs2517701 | 30033950 | class I | HLA-80 | HLA-80 pseudogene |
| rs6457109 | 30041239 | class I | HCG2P6 | HLA complex group 2 pseudogene 6 |
| rs4713270 | 30042675 | class I | HCG2P6 | HLA complex group 2 pseudogene 6 |
| rs4713274 | 30045471 | class I | MICD | MHC class I polypeptide-related sequence D |
| rs2256543 | 30045811 | class I | MICD | MHC class I polypeptide-related sequence D |
| rs435766 | 30047830 | class I | MICD | MHC class I polypeptide-related sequence D |
| rs2256820 | 30048084 | class I | MICD | MHC class I polypeptide-related sequence D |
| rs2256902 | 30048350 | class I | MICD | MHC class I polypeptide-related sequence D |
| rs2256919 | 30048728 | class I | MICD | MHC class I polypeptide-related sequence D |
| rs2523946 | 30049921 | class I | MICD | MHC class I polypeptide-related sequence D |
| rs3823355 | 30050061 | class I | MICD | MHC class I polypeptide-related sequence D |
| rs3823359 | 30050271 | class I | MICD | MHC class I polypeptide-related sequence D |
| rs422640 | 30050983 | class I |  |  |
| rs2245961 | 30051982 | class I | HCG9 | HLA complex group 9 |
| rs2571375 | 30053248 | class I | HCG9 | HLA complex group 9 |
| rs3132685 | 30053927 | class I | HCG9 | HLA complex group 9 |
| rs2246638 | 30057475 | class I | HLA-G | HLA-G histocompatibility antigen, class I, G |
| rs2394255 | 30057799 | class I | HLA-G | HLA-G histocompatibility antigen, class I, G |
| rs379221 | 30058118 | class I | HLA-G | HLA-G histocompatibility antigen, class I, G |
| rs401618 | 30058188 | class I | HLA-G | HLA-G histocompatibility antigen, class I, G |
| rs2735067 | 30059084 | class I | HLA-G | HLA-G histocompatibility antigen, class I, G |
| rs5025708 | 30063177 | class I | HLA-G | HLA-G histocompatibility antigen, class I, G |
| rs4959039 | 30065047 | class I | HLA-G | HLA-G histocompatibility antigen, class I, G |
| rs6911279 | 30073322 | class I | HLA-G | HLA-G histocompatibility antigen, class I, G |
| rs6904120 | 30075720 | class I | HLA-G | HLA-G histocompatibility antigen, class I, G |
| rs4713281 | 30086330 | class I | HLA-J | MHC, class I, J (pseudogene) |
| rs356971 | 30087775 | class I |  |  |
| rs9357092 | 30092230 | class I | HCG9 | HLA complex group 9 |
| rs9261145 | 30092843 | class I | HCG9 | HLA complex group 9 |
| rs3129012 | 30096620 | class I | HCG9 | HLA complex group 9 |
| rs165255 | 30097673 | class I | HCG9 | HLA complex group 9 |
| rs6919617 | 30099677 | class I | HCG9 | HLA complex group 9 |
| rs3121593 | 30110771 | class I | ETF1P1 | eukaryotic translation termination factor 1 pseudogene 1 |
| rs166327 | 30110859 | class I |  |  |
| rs259937 | 30115471 | class I |  |  |
| rs259939 | 30119559 | class I |  |  |
| rs259943 | 30123309 | class I |  |  |
| rs259919 | 30133481 | class I |  |  |
| rs3132130 | 30135430 | class I |  |  |
| rs3757329 | 30136402 | class I |  |  |
| rs3757333 | 30136584 | class I |  |  |
| rs7770505 | 30136891 | class I |  |  |
| rs7770557 | 30137087 | class I | ZNRD1 | zinc ribbon domain containing 1 |
| rs1150740 | 30138667 | class I | ZNRD1 | zinc ribbon domain containing 1 |
| rs9261290 | 30146625 | class I | RNF39 | ring finger protein 39 |
| rs9393989 | 30148062 | class I | RNF39 | ring finger protein 39 |
| rs2394734 | 30154224 | class I |  |  |
| rs6909253 | 30163621 | class I |  |  |
| rs1264708 | 30165132 | class I |  |  |
| rs11965797 | 30165741 | class I |  |  |
| rs9261387 | 30169339 | class I |  |  |
| rs1264703 | 30173394 | class I | TRIM31 | tripartite motif-containing 31 |
| rs1264697 | 30175546 | class I | TRIM31 | tripartite motif-containing 31 |
| rs2844795 | 30181825 | class I | TRIM31 | tripartite motif-containing 31 |
| rs2023472 | 30183842 | class I | TRIM31 | tripartite motif-containing 31 |
| rs2523990 | 30185207 | class I | TRIM31 | tripartite motif-containing 31 |
| rs4959041 | 30185945 | class I | TRIM31 | tripartite motif-containing 31 |
| rs2523988 | 30187107 | class I | TRIM31 | tripartite motif-containing 31 |
| rs3734838 | 30188209 | class I | TRIM31 | tripartite motif-containing 31 |
| rs2523984 | 30189981 | class I |  |  |
| rs1015465 | 30194318 | class I |  |  |
| rs9261440 | 30198798 | class I |  |  |
| rs1419675 | 30200685 | class I |  |  |
| rs1419673 | 30204695 | class I |  |  |
| rs757263 | 30206301 | class I |  |  |
| rs2523995 | 30210162 | class I |  |  |
| rs1541268 | 30211372 | class I | TRIM40 | tripartite motif-containing 40 |
| rs2021723 | 30211901 | class I | TRIM40 | tripartite motif-containing 40 |
| rs2107202 | 30213721 | class I | TRIM40 | tripartite motif-containing 40 |
| rs9261492 | 30218036 | class I | TRIM40 | tripartite motif-containing 40 |
| rs1557608 | 30226560 | class I | TRIM40 | tripartite motif-containing 40 |
| rs2517653 | 30229163 | class I | TRIM10 | tripartite motif-containing 10 |
| rs1008403 | 30240805 | class I | TRIM15 | tripartite motif-containing 15 |
| rs9368624 | 30243955 | class I | TRIM15 | tripartite motif-containing 15 |
| rs929157 | 30245187 | class I |  |  |
| rs2074475 | 30245868 | class I | TRIM15 | tripartite motif-containing 15 |
| rs1029239 | 30246140 | class I | TRIM15 | tripartite motif-containing 15 |
| rs757257 | 30250436 | class I |  |  |
| rs2844782 | 30258021 | class I |  |  |
| rs2239530 | 30260093 | class I | TRIM26 | tripartite motif-containing 26 |
| rs2517623 | 30260939 | class I | TRIM26 | tripartite motif-containing 26 |
| rs718254 | 30267017 | class I | TRIM26 | tripartite motif-containing 26 |
| rs2284165 | 30268033 | class I | TRIM26 | tripartite motif-containing 26 |
| rs2523721 | 30274244 | class I | TRIM26 | tripartite motif-containing 26 |
| rs2523720 | 30274864 | class I | TRIM26 | tripartite motif-containing 26 |
| rs2517610 | 30278258 | class I | TRIM26 | tripartite motif-containing 26 |
| rs2284168 | 30280211 | class I | TRIM26 | tripartite motif-containing 26 |
| rs3130383 | 30287004 | class I | TRIM26 | tripartite motif-containing 26 |
| rs2844775 | 30287400 | class I | TRIM26 | tripartite motif-containing 26 |
| rs3132667 | 30299565 | class I | TRIM26 | tripartite motif-containing 26 |
| rs885916 | 30310549 | class I |  |  |
| rs2844779 | 30313385 | class I |  |  |
| rs2253163 | 30317040 | class I |  |  |
| rs1362089 | 30317780 | class I |  |  |
| rs2523741 | 30319881 | class I | HLA-L | MHC, class I, L |
| rs3130404 | 30340228 | class I | FLJ45422 | FLJ45422 protein |
| rs2516698 | 30340352 | class I | FLJ45422 | FLJ45422 protein |
| rs1264624 | 30363067 | class I |  |  |
| rs10484551 | 30364816 | class I |  |  |
| rs1048664 | 30370490 | class I |  |  |
| rs261943 | 30381349 | class I |  |  |
| rs928822 | 30383224 | class I |  |  |
| rs9357097 | 30393099 | class I | TRIM39 | tripartite motif-containing 39 |
| rs1264582 | 30402568 | class I | TRIM39 | tripartite motif-containing 39 |
| rs1110464 | 30404049 | class I | TRIM39 | tripartite motif-containing 39 |
| rs3094064 | 30404231 | class I | TRIM39 | tripartite motif-containing 39 |
| rs3778624 | 30410578 | class I | TRIM39 | tripartite motif-containing 39 |
| rs3129838 | 30414531 | class I | TRIM39 | tripartite motif-containing 39 |
| rs3129840 | 30415320 | class I | TRIM39 | tripartite motif-containing 39 |
| rs2240058 | 30418478 | class I | TRIM39 | tripartite motif-containing 39 |
| rs974961 | 30422989 | class I | RPP21 | ribonuclease P/MRP 21kDa subunit |
| rs9380174 | 30424717 | class I | RPP21 | ribonuclease P/MRP 21kDa subunit |
| rs3094061 | 30429167 | class I | RPP21 | ribonuclease P/MRP 21kDa subunit |
| rs3130379 | 30433509 | class I |  |  |
| rs3094622 | 30435930 | class I |  |  |
| rs3130352 | 30436335 | class I |  |  |
| rs11754099 | 30437079 | class I |  |  |
| rs1012411 | 30440533 | class I |  |  |
| rs3129809 | 30443599 | class I |  |  |
| rs2022079 | 30445716 | class I |  |  |
| rs2844748 | 30446116 | class I |  |  |
| rs2523724 | 30447281 | class I |  |  |
| rs3130361 | 30447542 | class I |  |  |
| rs3129820 | 30451547 | class I |  |  |
| rs3132632 | 30452440 | class I |  |  |
| rs970270 | 30455284 | class I |  |  |
| rs7744914 | 30456225 | class I |  |  |
| rs2844743 | 30456810 | class I |  |  |
| rs3130109 | 30470729 | class I | PPP1R10 | protein phosphatase 1, regulatory (inhibitor) subunit 10 |
| rs2516688 | 30472502 | class I |  |  |
| rs1264569 | 30473298 | class I |  |  |
| rs1034325 | 30476435 | class I | DDR1 | discoidin domain receptor family, member 1 |
| rs1419689 | 30478566 | class I |  |  |
| rs1079991 | 30485838 | class I | TUBB | tubulin, beta |
| rs4713324 | 30488251 | class I | MICC | MHC class I polypeptide-related sequence C |
| rs1150770 | 30491934 | class I | MICC | MHC class I polypeptide-related sequence C |
| rs2074502 | 30492300 | class I | MICC | MHC class I polypeptide-related sequence C |
| rs1573294 | 30494393 | class I | MICC | MHC class I polypeptide-related sequence C |
| rs1264542 | 30494974 | class I | MICC | MHC class I polypeptide-related sequence C |
| rs9261947 | 30502606 | class I |  |  |
| rs1264524 | 30512764 | class I |  |  |
| rs9295863 | 30516243 | class I |  |  |
| rs1264511 | 30521950 | class I |  |  |
| rs1362115 | 30522826 | class I |  |  |
| rs2105961 | 30523614 | class I |  |  |
| rs2844729 | 30530610 | class I |  |  |
| rs2524180 | 30534108 | class I |  |  |
| rs2077573 | 30536329 | class I |  |  |
| rs2516676 | 30538460 | class I |  |  |
| rs3130141 | 30540155 | class I |  |  |
| rs2524172 | 30541589 | class I |  |  |
| rs7764934 | 30542544 | class I |  |  |
| rs9295895 | 30546204 | class I | LOC646520 | similar to succinate-CoA ligase, ADP-forming, beta subunit |
| rs2021720 | 30557222 | class I | RANP1 | RAN, member RAS oncogene family pseudogene 1 |
| rs2021719 | 30557460 | class I | RANP1 | RAN, member RAS oncogene family pseudogene 1 |
| rs1264459 | 30563898 | class I | RANP1 | RAN, member RAS oncogene family pseudogene 1 |
| rs2508024 | 30573490 | class I | HLA-E | MHC, class I, E |
| rs7754598 | 30575673 | class I | HLA-E | MHC, class I, E |
| rs3131115 | 30576769 | class I |  |  |
| rs2023609 | 30584012 | class I |  |  |
| rs975195 | 30585925 | class I |  |  |
| rs7768140 | 30596181 | class I |  |  |
| rs2524226 | 30605468 | class I |  |  |
| rs1468079 | 30614361 | class I |  |  |
| rs2524222 | 30619148 | class I |  |  |
| rs1058318 | 30620141 | class I |  |  |
| rs2074505 | 30629115 | class I | GNL1 | guanine nucleotide binding protein-like 1 |
| rs4713337 | 30637453 | class I |  |  |
| rs3888777 | 30637794 | class I | PRR3 | proline rich 3 |
| rs2074504 | 30638223 | class I | PRR3 | proline rich 3 |
| rs2074503 | 30638474 | class I | PRR3 | proline rich 3 |
| rs3132613 | 30645584 | class I | ABCF1 | ATP-binding cassette, sub-family F (GCN20), member 1 |
| rs2269709 | 30648868 | class I | ABCF1 | ATP-binding cassette, sub-family F (GCN20), member 1 |
| rs3132610 | 30652379 | class I | ABCF1 | ATP-binding cassette, sub-family F (GCN20), member 1 |
| rs6902544 | 30656188 | class I | ABCF1 | ATP-binding cassette, sub-family F (GCN20), member 1 |
| rs3130244 | 30660915 | class I | ABCF1 | ATP-binding cassette, sub-family F (GCN20), member 1 |
| rs1264432 | 30669999 | class I | ABCF1 | ATP-binding cassette, sub-family F (GCN20), member 1 |
| rs1264429 | 30673079 | class I |  |  |
| rs1264423 | 30679449 | class I | PPP1R10 | protein phosphatase 1, regulatory (inhibitor) subunit 10 |
| rs2267637 | 30686313 | class I | PPP1R10 | protein phosphatase 1, regulatory (inhibitor) subunit 10 |
| rs6457249 | 30687534 | class I | PPP1R10 | protein phosphatase 1, regulatory (inhibitor) subunit 10 |
| rs2517565 | 30702372 | class I | MRPS18B | mitochondrial ribosomal protein S18B |
| rs4713340 | 30707680 | class I | C6orf134 | chromosome 6 open reading frame 134 |
| rs2394390 | 30709799 | class I | PTMAP1 | prothymosin, α pseudogene 1 (gene sequence 26) |
| rs3132608 | 30717639 | class I | C6orf134 | chromosome 6 open reading frame 134 |
| rs1140809 | 30719654 | class I | C6orf134 | chromosome 6 open reading frame 134 |
| rs1076829 | 30735194 | class I | C6orf134 | chromosome 6 open reading frame 134 |
| rs4713346 | 30739041 | class I | DHX16 | DEAH (Asp-Glu-Ala-His) box polypeptide 16 |
| rs9295901 | 30747135 | class I | DHX16 | DEAH (Asp-Glu-Ala-His) box polypeptide 16 |
| rs9468805 | 30751687 | class I | KIAA1949 | KIAA1949 |
| rs2269707 | 30752502 | class I | KIAA1949 | KIAA1949 |
| rs6457254 | 30757112 | class I | KIAA1949 | KIAA1949 |
| rs3129996 | 30759565 | class I | KIAA1949 | KIAA1949 |
| rs1075496 | 30766217 | class I | NRM | nurim (nuclear envelope membrane protein) |
| rs9468811 | 30774647 | class I | MDC1 | mediator of DNA damage checkpoint 1 |
| rs2894043 | 30776467 | class I | MDC1 | mediator of DNA damage checkpoint 1 |
| rs2075015 | 30788586 | class I | MDC1 | mediator of DNA damage checkpoint 1 |
| rs9262152 | 30788894 | class I | MDC1 | mediator of DNA damage checkpoint 1 |
| rs4713354 | 30793398 | class I |  |  |
| rs3132584 | 30796405 | class I | TUBB | tubulin, beta |
| rs25527 | 30798916 | class I | TUBB | tubulin, beta |
| rs1064627 | 30806519 | class I | FLOT1 | flotillin 1 |
| rs1059612 | 30816933 | class I | FLOT1 | flotillin 1 |
| rs4713358 | 30824095 | class I | IER3 | immediate early response 3 |
| rs12210092 | 30824697 | class I |  |  |
| rs9262164 | 30825367 | class I |  |  |
| rs2535324 | 30826013 | class I |  |  |
| rs12202428 | 30827887 | class I |  |  |
| rs3129973 | 30829121 | class I |  |  |
| rs2394401 | 30832408 | class I |  |  |
| rs3131036 | 30836268 | class I |  |  |
| rs11966040 | 30837465 | class I |  |  |
| rs6926530 | 30838245 | class I |  |  |
| rs3094121 | 30838938 | class I |  |  |
| rs12526186 | 30844129 | class I |  |  |
| rs3130666 | 30848138 | class I |  |  |
| rs3130667 | 30851219 | class I |  |  |
| rs4713367 | 30864800 | class I |  |  |
| rs3131043 | 30866444 | class I |  |  |
| rs3131050 | 30868003 | class I |  |  |
| rs3094112 | 30869712 | class I |  |  |
| rs6930444 | 30871610 | class I |  |  |
| rs3130641 | 30872059 | class I |  |  |
| rs12660883 | 30872398 | class I |  |  |
| rs1264375 | 30874189 | class I |  |  |
| rs4711235 | 30876614 | class I |  |  |
| rs3129988 | 30877456 | class I |  |  |
| rs1264362 | 30884568 | class I |  |  |
| rs4327730 | 30888914 | class I |  |  |
| rs2394412 | 30890213 | class I |  |  |
| rs4713383 | 30895219 | class I |  |  |
| rs3094111 | 30896169 | class I |  |  |
| rs1264352 | 30897625 | class I |  |  |
| rs4947290 | 30902383 | class I |  |  |
| rs3130785 | 30904716 | class I |  |  |
| rs3095352 | 30913899 | class I |  |  |
| rs2394450 | 30925479 | class I |  |  |
| rs3130654 | 30931053 | class I |  |  |
| rs7761138 | 30938270 | class I |  |  |
| rs3095354 | 30944089 | class I |  |  |
| rs2535339 | 30947409 | class I |  |  |
| rs9461638 | 30959283 | class I | DDR1 | discoidin domain receptor family, member 1 |
| rs6457281 | 30965773 | class I | DDR1 |  |
| rs1264322 | 30965872 | class I | DDR1 | discoidin domain receptor family, member 1 |
| rs1264318 | 30971508 | class I | DDR1 | discoidin domain receptor family, member 1 |
| rs1049623 | 30972807 | class I | DDR1 | discoidin domain receptor family, member 1 |
| rs1049633 | 30975505 | class I | DDR1 | discoidin domain receptor family, member 1 |
| rs9468846 | 30978741 | class I | IER3 | immediate early response 3 |
| rs2894054 | 30980252 | class I | GTF2H4 | general transcription factor IIH, polypeptide 4, 52kDa |
| rs3130780 | 30982286 | class I | GTF2H4 | general transcription factor IIH, polypeptide 4, 52kDa |
| rs3218814 | 30986557 | class I | GTF2H4 | general transcription factor IIH, polypeptide 4, 52kDa |
| rs3218819 | 30988075 | class I | GTF2H4 | general transcription factor IIH, polypeptide 4, 52kDa |
| rs3218822 | 30988314 | class I | GTF2H4 | general transcription factor IIH, polypeptide 4, 52kDa |
| rs753725 | 30998849 | class I | VARSL | valyl-tRNA synthetase like |
| rs2252760 | 31000355 | class I | VARSL | valyl-tRNA synthetase like |
| rs2532940 | 31000570 | class I | VARSL | valyl-tRNA synthetase like |
| rs1043483 | 31001706 | class I | VARSL | valyl-tRNA synthetase like |
| rs4678 | 31001919 | class I | VARSL | valyl-tRNA synthetase like |
| rs2532934 | 31002737 | class I | VARSL | valyl-tRNA synthetase like |
| rs2253588 | 31007355 | class I | SFTPG | surfactant associated protein G |
| rs2286655 | 31007724 | class I | SFTPG | surfactant associated protein G |
| rs3131786 | 31009850 | class I |  |  |
| rs2844650 | 31010511 | class I |  |  |
| rs3132571 | 31013291 | class I |  |  |
| rs6933400 | 31015154 | class I |  |  |
| rs2517451 | 31022729 | class I |  |  |
| rs3130782 | 31022821 | class I |  |  |
| rs2247178 | 31024696 | class I |  |  |
| rs11970154 | 31027856 | class I | DPCR1 | diffuse panbronchiolitis critical region 1 |
| rs3132580 | 31028102 | class I | DPCR1 | diffuse panbronchiolitis critical region 1 |
| rs2240803 | 31028935 | class I | DPCR1 | diffuse panbronchiolitis critical region 1 |
| rs3131934 | 31039822 | class I | LOC646570 | hypothetical LOC646570 |
| rs3131783 | 31040046 | class I | LOC646570 | hypothetical LOC646570 |
| rs2530715 | 31041495 | class I | LOC646570 | hypothetical LOC646570 |
| rs3132579 | 31048967 | class I | C6orf15 | chromosome 6 open reading frame 15 |
| rs2844688 | 31050262 | class I |  |  |
| rs2517439 | 31050907 | class I |  |  |
| rs2844682 | 31054126 | class I |  |  |
| rs2517424 | 31057974 | class I | C6orf15 | chromosome 6 open reading frame 15 |
| rs6918439 | 31059756 | class I | C6orf205 | chromosome 6 open reading frame 205 |
| rs2517415 | 31064757 | class I | C6orf205 | chromosome 6 open reading frame 205 |
| rs3873342 | 31065744 | class I | C6orf205 | chromosome 6 open reading frame 205 |
| rs2517408 | 31074431 | class I |  |  |
| rs2530690 | 31075180 | class I |  |  |
| rs11964080 | 31078948 | class I |  |  |
| rs1634717 | 31080567 | class I |  |  |
| rs1634718 | 31080843 | class I |  |  |
| rs3869095 | 31087993 | class I |  |  |
| rs7755364 | 31089693 | class I |  |  |
| rs6903912 | 31099537 | class I |  |  |
| rs12198448 | 31100324 | class I |  |  |
| rs2523897 | 31101936 | class I | LOC729792 | hypothetical protein LOC729792 |
| rs9262549 | 31105670 | class I | LOC729792 | hypothetical protein LOC729792 |
| rs6457300 | 31106720 | class I | LOC729792 | hypothetical protein LOC729792 |
| rs4713422 | 31107880 | class I | LOC729792 | hypothetical protein LOC729792 |
| rs9468862 | 31109333 | class I | LOC729792 | hypothetical protein LOC729792 |
| rs2844665 | 31114833 | class I | LOC729792 | hypothetical protein LOC729792 |
| rs2517545 | 31117280 | class I | LOC729792 | hypothetical protein LOC729792 |
| rs12528584 | 31119823 | class I | LOC729792 | hypothetical protein LOC729792 |
| rs2523870 | 31122094 | class I | LOC729792 | hypothetical protein LOC729792 |
| rs2844645 | 31123160 | class I |  |  |
| rs2523864 | 31126524 | class I |  |  |
| rs4713429 | 31128995 | class I |  |  |
| rs3764808 | 31132651 | class I |  |  |
| rs2523849 | 31133029 | class I |  |  |
| rs9262636 | 31133826 | class I |  |  |
| rs2523841 | 31138261 | class I |  |  |
| rs2517500 | 31141487 | class I |  |  |
| rs2523881 | 31150586 | class I |  |  |
| rs2106067 | 31156241 | class I |  |  |
| rs6941772 | 31156894 | class I |  |  |
| rs9380215 | 31157633 | class I |  |  |
| rs2517471 | 31160076 | class I |  |  |
| rs2535310 | 31161235 | class I |  |  |
| rs3130955 | 31162489 | class I |  |  |
| rs4947296 | 31166156 | class I |  |  |
| rs2517448 | 31170645 | class I |  |  |
| rs4713433 | 31176005 | class I |  |  |
| rs6457327 | 31182008 | class I |  |  |
| rs2233984 | 31187242 | class I | C6orf15 | chromosome 6 open reading frame 15 |
| rs2233974 | 31187994 | class I | C6orf15 | chromosome 6 open reading frame 15 |
| rs2270190 | 31188564 | class I | CDSN | corneodesmosin |
| rs2233956 | 31189183 | class I |  |  |
| rs3130977 | 31189967 | class I | PSORS1C1 | psoriasis susceptibility 1 candidate 1 |
| rs3130552 | 31190105 | class I | PSORS1C1 | psoriasis susceptibility 1 candidate 1 |
| rs1042141 | 31191444 | class I | PSORS1C1 | psoriasis susceptibility 1 candidate 1 |
| rs3094216 | 31192026 | class I | PSORS1C1 | psoriasis susceptibility 1 candidate 1 |
| rs1062470 | 31192413 | class I | PSORS1C1 | psoriasis susceptibility 1 candidate 1 |
| rs4713436 | 31192617 | class I | PSORS1C1 | psoriasis susceptibility 1 candidate 1 |
| rs3132550 | 31194026 | class I | PSORS1C1 | psoriasis susceptibility 1 candidate 1 |
| rs2239519 | 31196834 | class I | PSORS1C1 | psoriasis susceptibility 1 candidate 1 |
| rs2239523 | 31197485 | class I | PSORS1C1 | psoriasis susceptibility 1 candidate 1 |
| rs3095313 | 31198577 | class I | PSORS1C1 | psoriasis susceptibility 1 candidate 1 |
| rs3095307 | 31200027 | class I | PSORS1C1 | psoriasis susceptibility 1 candidate 1 |
| rs3778638 | 31200102 | class I | PSORS1C1 | psoriasis susceptibility 1 candidate 1 |
| rs3095302 | 31201044 | class I | PSORS1C1 | psoriasis susceptibility 1 candidate 1 |
| rs3823418 | 31208920 | class I | PSORS1C1 | psoriasis susceptibility 1 candidate 1 |
| rs4084091 | 31209379 | class I | PSORS1C1 | psoriasis susceptibility 1 candidate 1 |
| rs1265100 | 31213288 | class I | PSORS1C1 | psoriasis susceptibility 1 candidate 1 |
| rs2074478 | 31213611 | class I | PSORS1C2 | psoriasis susceptibility 1 candidate 2 |
| rs1265087 | 31217788 | class I | CCHCR1 | coiled-coil α-helical rod protein 1 |
| rs1265085 | 31218649 | class I | CCHCR1 | coiled-coil α-helical rod protein 1 |
| rs1265078 | 31220580 | class I | CCHCR1 | coiled-coil α-helical rod protein 1 |
| rs3094225 | 31221030 | class I | CCHCR1 | coiled-coil α-helical rod protein 1 |
| rs2240066 | 31222313 | class I | CCHCR1 | coiled-coil α-helical rod protein 1 |
| rs3131012 | 31223419 | class I | CCHCR1 | coiled-coil α-helical rod protein 1 |
| rs1265115 | 31225053 | class I | CCHCR1 | coiled-coil α-helical rod protein 1 |
| rs130078 | 31226543 | class I | CCHCR1 | coiled-coil α-helical rod protein 1 |
| rs2073716 | 31230975 | class I | CCHCR1 | coiled-coil α-helical rod protein 1 |
| rs3130453 | 31232827 | class I | CCHCR1 | coiled-coil α-helical rod protein 1 |
| rs3094187 | 31234922 | class I | TCF19 | transcription factor 19 (SC1) |
| rs1150765 | 31235540 | class I | TCF19 | transcription factor 19 (SC1) |
| rs2073724 | 31237685 | class I | TCF19 | transcription factor 19 (SC1) |
| rs1065461 | 31238480 | class I | TCF19 | transcription factor 19 (SC1) |
| rs13409 | 31240118 | class I | POU5F1 | POU class 5 homeobox 1 |
| rs2106074 | 31241487 | class I | POU5F1 | POU class 5 homeobox 1 |
| rs3130931 | 31242866 | class I | POU5F1 | POU class 5 homeobox 1 |
| rs3130501 | 31244431 | class I | POU5F1 | POU class 5 homeobox 1 |
| rs3132524 | 31244692 | class I | POU5F1 | POU class 5 homeobox 1 |
| rs879882 | 31247430 | class I | POU5F1 | POU class 5 homeobox 1 |
| rs885948 | 31248510 | class I | POU5F1 | POU class 5 homeobox 1 |
| rs1265158 | 31248719 | class I |  |  |
| rs887468 | 31249501 | class I |  |  |
| rs887466 | 31251489 | class I |  |  |
| rs3131018 | 31251560 | class I |  |  |
| rs915660 | 31251813 | class I |  |  |
| rs3871248 | 31253969 | class I |  |  |
| rs1052989 | 31254381 | class I |  |  |
| rs3130457 | 31255172 | class I |  |  |
| rs1265181 | 31263763 | class I |  |  |
| rs4713447 | 31270941 | class I | HCG27 | HLA complex group 27 |
| rs3094609 | 31273544 | class I | HCG27 | HLA complex group 27 |
| rs4122189 | 31275905 | class I | HCG27 | HLA complex group 27 |
| rs9263873 | 31278691 | class I | HCG27 | HLA complex group 27 |
| rs2394885 | 31282569 | class I |  |  |
| rs9263964 | 31294017 | class I |  |  |
| rs1345274 | 31298605 | class I |  |  |
| rs12662501 | 31298828 | class I |  |  |
| rs6904669 | 31300774 | class I |  |  |
| rs7745906 | 31311986 | class I |  |  |
| rs3130685 | 31314184 | class I |  |  |
| rs3134768 | 31314822 | class I |  |  |
| rs2844623 | 31340521 | class I | HLA-C | MHC, class I, C |
| rs2394953 | 31341331 | class I |  |  |
| rs2074488 | 31348409 | class I | HLA-C | MHC, class I, C |
| rs3132486 | 31351148 | class I |  |  |
| rs2524074 | 31351999 | class I |  |  |
| rs2524069 | 31352767 | class I |  |  |
| rs7381988 | 31354681 | class I |  |  |
| rs7382297 | 31355045 | class I |  |  |
| rs9461684 | 31361422 | class I |  |  |
| rs2853933 | 31362066 | class I |  |  |
| rs2524044 | 31364731 | class I |  |  |
| rs2524040 | 31365603 | class I |  |  |
| rs2256583 | 31366824 | class I |  |  |
| rs2524163 | 31367557 | class I |  |  |
| rs2524160 | 31367832 | class I |  |  |
| rs3873379 | 31370147 | class I |  |  |
| rs2853926 | 31371029 | class I |  |  |
| rs2853925 | 31372900 | class I |  |  |
| rs3905495 | 31373517 | class I |  |  |
| rs2524089 | 31374500 | class I |  |  |
| rs6457374 | 31380239 | class I |  |  |
| rs7760988 | 31381008 | class I |  |  |
| rs2524229 | 31383209 | class I |  |  |
| rs2923006 | 31425077 | class I |  |  |
| rs2844586 | 31426002 | class I |  |  |
| rs2523619 | 31426122 | class I |  |  |
| rs2442719 | 31428516 | class I | HLA-B | MHC, class I, B |
| rs2596501 | 31429189 | class I | HLA-B | MHC, class I, B |
| rs3819294 | 31430465 | class I | HLA-B | MHC, class I, B |
| rs2523589 | 31435312 | class I | HLA-B | MHC, class I, B |
| rs2523554 | 31439807 | class I |  |  |
| rs2844580 | 31441281 | class I |  |  |
| rs2844573 | 31443432 | class I | LOC729816 | similar to Dihydrofolate reductase |
| rs2596438 | 31447849 | class I |  |  |
| rs2254556 | 31450609 | class I |  |  |
| rs6933050 | 31451610 | class I |  |  |
| rs2507983 | 31453772 | class I |  |  |
| rs2442738 | 31454392 | class I |  |  |
| rs2844542 | 31455252 | class I |  |  |
| rs2844533 | 31458780 | class I |  |  |
| rs2442752 | 31459742 | class I |  |  |
| rs2853969 | 31464552 | class I |  |  |
| rs2523467 | 31470908 | class I |  |  |
| rs2523451 | 31477129 | class I | MICA | MHC class I polypeptide-related sequence A |
| rs1051794 | 31487087 | class I | MICA | MHC class I polypeptide-related sequence A |
| rs2848716 | 31495945 | class I |  |  |
| rs2844511 | 31497762 | class I |  |  |
| rs6915220 | 31515799 | class I |  |  |
| rs2524276 | 31516243 | class I |  |  |
| rs2523693 | 31526102 | class I |  |  |
| rs2516436 | 31527855 | class I |  |  |
| rs2596480 | 31533963 | class I |  |  |
| rs2596472 | 31536945 | class I |  |  |
| rs2596473 | 31538777 | class I | HCP5 | HLA complex P5 |
| rs2395029 | 31539758 | class I | HCP5 | HLA complex P5 |
| rs2244546 | 31543811 | class I | HCP5 | HLA complex P5 |
| rs2859448 | 31544251 | class I |  |  |
| rs2844508 | 31544477 | class I |  |  |
| rs2523674 | 31544767 | class I |  |  |
| rs2395031 | 31545283 | class I |  |  |
| rs2596449 | 31546368 | class I | 3.8-1 | MHC class I mRNA fragment 3.8-1 |
| rs2516440 | 31548475 | class I |  |  |
| rs6940467 | 31550115 | class I |  |  |
| rs3131643 | 31550760 | class I |  |  |
| rs2523656 | 31552026 | class I |  |  |
| rs2248462 | 31554774 | class I |  |  |
| rs2596536 | 31555826 | class I |  |  |
| rs2904776 | 31557059 | class I |  |  |
| rs2523647 | 31557756 | class I |  |  |
| rs3130922 | 31569067 | class I |  |  |
| rs3828901 | 31571696 | class I | MICB | MHC class I polypeptide-related sequence B |
| rs2534673 | 31573468 | class I | MICB | MHC class I polypeptide-related sequence B |
| rs3131635 | 31584112 | class I | MICB | MHC class I polypeptide-related sequence B |
| rs3095229 | 31588250 | class III |  |  |
| rs2516398 | 31589504 | class III |  |  |
| rs2844494 | 31591393 | class III |  |  |
| rs4959079 | 31596857 | class III | PPIAP9 | peptidylprolyl isomerase A (cyclophilin A) pseudogene 9 |
| rs3132454 | 31597622 | class III |  |  |
| rs3093993 | 31598703 | class III |  |  |
| rs3095227 | 31598978 | class III |  |  |
| rs3093986 | 31601399 | class III |  |  |
| rs3130055 | 31605377 | class III |  |  |
| rs3093978 | 31606475 | class III | BAT1 | HLA-B associated transcript 1 |
| rs3131628 | 31610745 | class III | BAT1 | HLA-B associated transcript 1 |
| rs2071596 | 31614669 | class III | BAT1 | HLA-B associated transcript 1 |
| rs2523512 | 31614779 | class III | BAT1 | HLA-B associated transcript 1 |
| rs2239709 | 31615425 | class III | BAT1 | HLA-B associated transcript 1 |
| rs3130059 | 31617262 | class III | BAT1 | HLA-B associated transcript 1 |
| rs2239528 | 31618083 | class III | BAT1 | HLA-B associated transcript 1 |
| rs2071594 | 31620698 | class III | ATP6V1G2 | ATPase, H+ transporting, lysosomal 13kDa, V1 subunit G2 |
| rs2239705 | 31621380 | class III | ATP6V1G2 | ATPase, H+ transporting, lysosomal 13kDa, V1 subunit G2 |
| rs2523503 | 31621537 | class III | ATP6V1G2 | ATPase, H+ transporting, lysosomal 13kDa, V1 subunit G2 |
| rs2071591 | 31623777 | class III | NFKBIL1 | nuclear factor of κ light polypeptide gene enhancer in B-cells inhibitor-like 1 |
| rs3219183 | 31624341 | class III | NFKBIL1 | nuclear factor of κ light polypeptide gene enhancer in B-cells inhibitor-like 1 |
| rs2255798 | 31629280 | class III | NFKBIL1 | nuclear factor of κ light polypeptide gene enhancer in B-cells inhibitor-like 1 |
| rs6929796 | 31630647 | class III | NFKBIL1 | nuclear factor of κ light polypeptide gene enhancer in B-cells inhibitor-like 1 |
| rs2857605 | 31632829 | class III | NFKBIL1 | nuclear factor of κ light polypeptide gene enhancer in B-cells inhibitor-like 1 |
| rs2239707 | 31633297 | class III | NFKBIL1 | nuclear factor of κ light polypeptide gene enhancer in B-cells inhibitor-like 1 |
| rs4947324 | 31636108 | class III | NFKBIL1 | nuclear factor of κ light polypeptide gene enhancer in B-cells inhibitor-like 1 |
| rs7762619 | 31639288 | class III |  |  |
| rs2844484 | 31644202 | class III |  |  |
| rs2516312 | 31647413 | class III | LTA | lymphotoxin α (TNF superfamily, member 1) |
| rs1799964 | 31650286 | class III | LTA | lymphotoxin α (TNF superfamily, member 1) |
| rs1800629 | 31651009 | class III | TNF | tumor necrosis factor (TNF superfamily, member 2) |
| rs3093661 | 31651736 | class III | TNF | tumor necrosis factor (TNF superfamily, member 2) |
| rs3093662 | 31652167 | class III | TNF | tumor necrosis factor (TNF superfamily, member 2) |
| rs4645843 | 31652540 | class III | TNF | tumor necrosis factor (TNF superfamily, member 2) |
| rs3093559 | 31655770 | class III |  |  |
| rs2844480 | 31672799 | class III | NCR3 | natural cytotoxicity triggering receptor 3 |
| rs2857596 | 31675400 | class III |  |  |
| rs2857595 | 31676447 | class III |  |  |
| rs2844479 | 31680934 | class III |  |  |
| rs3132451 | 31690003 | class III |  |  |
| rs2736176 | 31695539 | class III |  |  |
| rs2857694 | 31695848 | class III | BAT2 | HLA-B associated transcript 2 |
| rs2857693 | 31696362 | class III | BAT2 | HLA-B associated transcript 2 |
| rs2844472 | 31697654 | class III | BAT2 | HLA-B associated transcript 2 |
| rs3130622 | 31700502 | class III | SNORA38 | small nucleolar RNA, H/ACA box 38 |
| rs3130071 | 31702606 | class III |  |  |
| rs2736171 | 31703465 | class III |  |  |
| rs2242660 | 31705731 | class III |  |  |
| rs2736161 | 31706271 | class III |  |  |
| rs10885 | 31712569 | class III |  |  |
| rs2736155 | 31713177 | class III | BAT3 | HLA-B associated transcript 3 |
| rs2242656 | 31722080 | class III | BAT3 | HLA-B associated transcript 3 |
| rs805301 | 31726099 | class III | BAT3 | HLA-B associated transcript 3 |
| rs707921 | 31733519 | class III | APOM | apolipoprotein M |
| rs805263 | 31736091 | class III | C6orf47 | chromosome 6 open reading frame 47 |
| rs805262 | 31736711 | class III | C6orf47 | chromosome 6 open reading frame 47 |
| rs11965323 | 31737616 | class III | BAT4 | HLA-B associated transcript 4 |
| rs7992 | 31738219 | class III | BAT4 | HLA-B associated transcript 4 |
| rs9267532 | 31747957 | class III | LY6G5B | lymphocyte antigen 6 complex, locus G5B |
| rs2280800 | 31754376 | class III | LY6G5C | lymphocyte antigen 6 complex, locus G5C |
| rs805274 | 31773172 | class III | BAT5 | HLA-B associated transcript 5 |
| rs1266074 | 31774529 | class III | BAT5 | HLA-B associated transcript 5 |
| rs2242653 | 31783743 | class III | C6orf21 | chromosome 6 open reading frame 21 |
| rs805285 | 31787443 | class III | LY6G6E | lymphocyte antigen 6 complex, locus G6E |
| rs4713479 | 31796777 | class III | LY6G6C | lymphocyte antigen 6 complex, locus G6C |
| rs805292 | 31797987 | class III | LY6G6C | lymphocyte antigen 6 complex, locus G6C |
| rs453098 | 31799635 | class III | C6orf25 | chromosome 6 open reading frame 25 |
| rs2272592 | 31806330 | class III | DDAH2 | dimethylarginine dimethylaminohydrolase 2 |
| rs400547 | 31810688 | class III | CLIC1 | chloride intracellular channel 1 |
| rs3131382 | 31815708 | class III | MSH5 | mutS homolog 5 (E. coli) |
| rs707915 | 31818946 | class III | MSH5 | mutS homolog 5 (E. coli) |
| rs3749953 | 31821102 | class III | MSH5 | mutS homolog 5 (E. coli) |
| rs3828922 | 31821432 | class III | MSH5 | mutS homolog 5 (E. coli) |
| rs3130484 | 31823860 | class III | MSH5 | mutS homolog 5 (E. coli) |
| rs3131379 | 31829011 | class III | MSH5 | mutS homolog 5 (E. coli) |
| rs3131378 | 31833263 | class III | MSH5 | mutS homolog 5 (E. coli) |
| rs707936 | 31841628 | class III | C6orf27 | chromosome 6 open reading frame 27 |
| rs7769537 | 31849959 | class III | C6orf27 | chromosome 6 open reading frame 27 |
| rs707929 | 31850045 | class III | C6orf27 | chromosome 6 open reading frame 27 |
| rs7769899 | 31850219 | class III | C6orf27 | chromosome 6 open reading frame 27 |
| rs707926 | 31856798 | class III | VARS | valyl-tRNA synthetase |
| rs4713488 | 31870289 | class III | VARS | valyl-tRNA synthetase |
| rs2227955 | 31886055 | class III | HSPA1L | heat shock 70kDa protein 1-like |
| rs2075799 | 31886507 | class III |  |  |
| rs2763979 | 31902570 | class III | HSPA1B | heat shock 70kDa protein 1B |
| rs2471980 | 31908846 | class III | HSPA1B | heat shock 70kDa protein 1B |
| rs11968400 | 31912707 | class III | SNORD48 | small nucleolar RNA, C/D box 48 |
| rs9469063 | 31921554 | class III | SNORD52 | small nucleolar RNA, C/D box 52 |
| rs12210887 | 31923701 | class III |  |  |
| rs9267649 | 31932806 | class III | NEU1 | sialidase 1 (lysosomal sialidase) |
| rs13118 | 31935264 | class III | NEU1 | sialidase 1 (lysosomal sialidase) |
| rs6915800 | 31941638 | class III | SLC44A4 | solute carrier family 44, member 4 |
| rs4947332 | 31942175 | class III | SLC44A4 | solute carrier family 44, member 4 |
| rs660550 | 31945255 | class III | SLC44A4 | solute carrier family 44, member 4 |
| rs644827 | 31946419 | class III | SLC44A4 |  |
| rs3130481 | 31947734 | class III | SLC44A4 | solute carrier family 44, member 4 |
| rs614549 | 31948603 | class III | SLC44A4 | solute carrier family 44, member 4 |
| rs2736428 | 31951902 | class III | SLC44A4 | solute carrier family 44, member 4 |
| rs605203 | 31954990 | class III | EHMT2 | euchromatic histone-lysine N-methyltransferase 2 |
| rs652888 | 31959212 | class III | EHMT2 | euchromatic histone-lysine N-methyltransferase 2 |
| rs659445 | 31972282 | class III | EHMT2 | euchromatic histone-lysine N-methyltransferase 2 |
| rs558702 | 31978304 | class III | ZBTB12 | zinc finger and BTB domain containing 12 |
| rs2763982 | 31980529 | class III |  |  |
| rs644045 | 31991935 | class III |  |  |
| rs544167 | 31998136 | class III |  |  |
| rs2734335 | 32001922 | class III |  |  |
| rs7746553 | 32003951 | class III | C2 | complement component 2 |
| rs9332730 | 32019987 | class III | C2 | complement component 2 |
| rs4151651 | 32023592 | class III | CFB | complement factor B |
| rs2072634 | 32025269 | class III | CFB | complement factor B |
| rs4151657 | 32025518 | class III | CFB | complement factor B |
| rs4151659 | 32026442 | class III | CFB | complement factor B |
| rs4151664 | 32028851 | class III | RDBP | RD RNA binding protein |
| rs630379 | 32030232 | class III | RDBP | RD RNA binding protein |
| rs440454 | 32035320 | class III | SKIV2L | superkiller viralicidic activity 2-like (S. cerevisiae) |
| rs437179 | 32036992 | class III | SKIV2L | superkiller viralicidic activity 2-like (S. cerevisiae) |
| rs2734331 | 32038329 | class III | SKIV2L | superkiller viralicidic activity 2-like (S. cerevisiae) |
| rs429608 | 32038440 | class III | SKIV2L | superkiller viralicidic activity 2-like (S. cerevisiae) |
| rs474534 | 32046085 | class III | DOM3Z | dom-3 homolog Z (C. elegans) |
| rs389883 | 32055438 | class III | STK19 | serine/threonine kinase 19 |
| rs389512 | 32055572 | class III | STK19 | serine/threonine kinase 19 |
| rs1150758 | 32136126 | class III | TNXB | tenascin XB |
| rs2269429 | 32137160 | class III | TNXB | tenascin XB |
| rs2071295 | 32146677 | class III | TNXB | tenascin XB |
| rs204879 | 32151134 | class III | TNXB | tenascin XB |
| rs7774197 | 32154252 | class III | TNXB | tenascin XB |
| rs204900 | 32164557 | class III | TNXB | tenascin XB |
| rs2071293 | 32170664 | class III | TNXB | tenascin XB |
| rs3134954 | 32179870 | class III | TNXB | tenascin XB |
| rs2269426 | 32184476 | class III | TNXB | tenascin XB |
| rs411337 | 32185357 | class III | TNXB | tenascin XB |
| rs1269852 | 32188168 | class III | CREBL1 | cAMP responsive element binding protein-like 1 |
| rs8111 | 32191152 | class III | CREBL1 | cAMP responsive element binding protein-like 1 |
| rs204890 | 32193575 | class III | CREBL1 | cAMP responsive element binding protein-like 1 |
| rs204888 | 32197119 | class III | CREBL1 | cAMP responsive element binding protein-like 1 |
| rs3830076 | 32204221 | class III | FKBPL | FK506 binding protein like |
| rs169494 | 32205853 | class III | FKBPL | FK506 binding protein like |
| rs4713505 | 32212978 | class III | PRRT1 | proline-rich transmembrane protein 1 |
| rs204999 | 32217956 | class III | FKBPL | FK506 binding protein like |
| rs3131283 | 32227875 | class III | PRRT1 | proline-rich transmembrane protein 1 |
| rs3134950 | 32235454 | class III | PPT2 | palmitoyl-protein thioesterase 2 |
| rs205000 | 32243855 | class III | EGFL8 | EGF-like-domain, multiple 8 |
| rs3130283 | 32246522 | class III | AGPAT1 | 1-acylglycerol-3-phosphate O-acyltransferase 1 (lysophosphatidic acid acyltransferase, α) |
| rs3131297 | 32248982 | class III | AGPAT1 | 1-acylglycerol-3-phosphate O-acyltransferase 1 (lysophosphatidic acid acyltransferase, α) |
| rs3134945 | 32254469 | class III | RNF5 | ring finger protein 5 |
| rs3130349 | 32255673 | class III | RNF5 | ring finger protein 5 |
| rs204996 | 32257860 | class III | AGER | advanced glycosylation end product-specific receptor |
| rs1800625 | 32260419 | class III | PBX2 | pre-B-cell leukemia homeobox 2 |
| rs204994 | 32262975 | class III | PBX2 | pre-B-cell leukemia homeobox 2 |
| rs204992 | 32264885 | class III | PBX2 | pre-B-cell leukemia homeobox 2 |
| rs176095 | 32266296 | class III | GPSM3 | G-protein signaling modulator 3 (AGS3-like, C. elegans) |
| rs204991 | 32269343 | class III | GPSM3 | G-protein signaling modulator 3 (AGS3-like, C. elegans) |
| rs8192579 | 32271776 | class III | NOTCH4 | Notch homolog 4 (Drosophila) |
| rs2071278 | 32273421 | class III | NOTCH4 | Notch homolog 4 (Drosophila) |
| rs3134942 | 32276748 | class III | NOTCH4 | Notch homolog 4 (Drosophila) |
| rs3132935 | 32279052 | class III | NOTCH4 | Notch homolog 4 (Drosophila) |
| rs2071277 | 32279660 | class III | NOTCH4 | Notch homolog 4 (Drosophila) |
| rs1044506 | 32280042 | class III | NOTCH4 | Notch homolog 4 (Drosophila) |
| rs206018 | 32285857 | class III | NOTCH4 | Notch homolog 4 (Drosophila) |
| rs2071285 | 32288408 | class III | NOTCH4 | Notch homolog 4 (Drosophila) |
| rs206015 | 32290736 | class III | NOTCH4 | Notch homolog 4 (Drosophila) |
| rs384247 | 32292551 | class III | NOTCH4 | Notch homolog 4 (Drosophila) |
| rs379464 | 32294325 | class III | NOTCH4 | Notch homolog 4 (Drosophila) |
| rs394657 | 32295000 | class III | NOTCH4 | Notch homolog 4 (Drosophila) |
| rs423023 | 32296274 | class III | NOTCH4 | Notch homolog 4 (Drosophila) |
| rs3134931 | 32298597 | class III | NOTCH4 | Notch homolog 4 (Drosophila) |
| rs3830041 | 32299316 | class III | NOTCH4 | Notch homolog 4 (Drosophila) |
| rs396960 | 32299558 | class III | NOTCH4 | Notch homolog 4 (Drosophila) |
| rs397596 | 32300461 | class II |  |  |
| rs2267644 | 32300537 | class II |  |  |
| rs1475961 | 32302586 | class II |  |  |
| rs3096691 | 32302831 | class II |  |  |
| rs365053 | 32303965 | class II |  |  |
| rs3130299 | 32311514 | class II |  |  |
| rs416352 | 32315370 | class II |  |  |
| rs424232 | 32316301 | class II |  |  |
| rs382259 | 32317004 | class II |  |  |
| rs507778 | 32317838 | class II |  |  |
| rs419132 | 32318776 | class II |  |  |
| rs440169 | 32321765 | class II |  |  |
| rs6936204 | 32325069 | class II |  |  |
| rs3130315 | 32328662 | class II |  |  |
| rs3130316 | 32329205 | class II | LOC401252 | hypothetical gene supported by AK123889 |
| rs3096700 | 32329759 | class II | LOC401252 | hypothetical gene supported by AK123889 |
| rs3115569 | 32332117 | class II | LOC401252 | hypothetical gene supported by AK123889 |
| rs926070 | 32365543 | class II | TSBP | Testes Specific Basic Protein |
| rs9268148 | 32367504 | class II | TSBP | Testes Specific Basic Protein |
| rs3749966 | 32369484 | class II | TSBP | Testes Specific Basic Protein |
| rs477005 | 32378477 | class II | TSBP | Testes Specific Basic Protein |
| rs7341328 | 32383171 | class II | TSBP | Testes Specific Basic Protein |
| rs6910071 | 32390831 | class II | TSBP | Testes Specific Basic Protein |
| rs3132958 | 32405878 | class II | TSBP | Testes Specific Basic Protein |
| rs3129949 | 32406791 | class II | TSBP | Testes Specific Basic Protein |
| rs3129904 | 32418373 | class II | TSBP | Testes Specific Basic Protein |
| rs1555117 | 32419125 | class II | TSBP | Testes Specific Basic Protein |
| rs6904320 | 32420059 | class II | TSBP | Testes Specific Basic Protein |
| rs2076540 | 32425253 | class II | TSBP | Testes Specific Basic Protein |
| rs2076537 | 32425612 | class II | TSBP | Testes Specific Basic Protein |
| rs3132963 | 32428130 | class II | TSBP | Testes Specific Basic Protein |
| rs1265759 | 32430370 | class II | TSBP | Testes Specific Basic Protein |
| rs6907322 | 32432922 | class II | TSBP | Testes Specific Basic Protein |
| rs2273017 | 32445607 | class II | TSBP | Testes Specific Basic Protein |
| rs3129943 | 32446672 | class II | TSBP | Testes Specific Basic Protein |
| rs2050191 | 32446878 | class II | TSBP | Testes Specific Basic Protein |
| rs2073044 | 32446963 | class II | TSBP | Testes Specific Basic Protein |
| rs2050190 | 32447053 | class II | TSBP | Testes Specific Basic Protein |
| rs2076536 | 32447325 | class II | TSBP | Testes Specific Basic Protein |
| rs2076535 | 32447488 | class II | TSBP | Testes Specific Basic Protein |
| rs2050189 | 32447624 | class II | TSBP | Testes Specific Basic Protein |
| rs2395153 | 32453572 | class II |  |  |
| rs1980495 | 32454771 | class II |  |  |
| rs1555115 | 32462497 | class II |  |  |
| rs3117098 | 32466490 | class II |  |  |
| rs3817973 | 32469088 | class II | BTNL2 | butyrophilin-like 2 (MHC class II associated) |
| rs3817969 | 32469365 | class II | BTNL2 | butyrophilin-like 2 (MHC class II associated) |
| rs1980493 | 32471192 | class II | BTNL2 | butyrophilin-like 2 (MHC class II associated) |
| rs2076530 | 32471793 | class II | BTNL2 | butyrophilin-like 2 (MHC class II associated) |
| rs2294884 | 32475236 | class II | BTNL2 | butyrophilin-like 2 (MHC class II associated) |
| rs2294883 | 32475428 | class II | BTNL2 | butyrophilin-like 2 (MHC class II associated) |
| rs3817966 | 32475824 | class II | BTNL2 | butyrophilin-like 2 (MHC class II associated) |
| rs3817964 | 32475974 | class II | BTNL2 | butyrophilin-like 2 (MHC class II associated) |
| rs2076520 | 32479246 | class II | BTNL2 | butyrophilin-like 2 (MHC class II associated) |
| rs3806156 | 32481675 | class II | BTNL2 | butyrophilin-like 2 (MHC class II associated) |
| rs3806157 | 32481778 | class II | BTNL2 | butyrophilin-like 2 (MHC class II associated) |
| rs6926737 | 32483722 | class II | BTNL2 | butyrophilin-like 2 (MHC class II associated) |
| rs9268507 | 32485516 | class II |  |  |
| rs3129961 | 32486917 | class II |  |  |
| rs2187820 | 32493850 | class II |  |  |
| rs2395165 | 32496121 | class II |  |  |
| rs3135366 | 32496686 | class II |  |  |
| rs3135363 | 32497625 | class II |  |  |
| rs3135352 | 32500883 | class II |  |  |
| rs6457580 | 32501118 | class II |  |  |
| rs2395171 | 32502514 | class II |  |  |
| rs2187818 | 32503545 | class II |  |  |
| rs984778 | 32508065 | class II |  |  |
| rs3129859 | 32508916 | class II |  |  |
| rs3135338 | 32509194 | class II |  |  |
| rs7773756 | 32510441 | class II |  |  |
| rs2395175 | 32513003 | class II | HLA-DRA | MHC, class II, DR α |
| rs2395178 | 32513339 | class II | HLA-DRA | MHC, class II, DR α |
| rs14004 | 32515686 | class II | HLA-DRA | MHC, class II, DR α |
| rs3129876 | 32515989 | class II | HLA-DRA | MHC, class II, DR α |
| rs3129878 | 32516712 | class II | HLA-DRA | MHC, class II, DR α |
| rs3129882 | 32517507 | class II | HLA-DRA | MHC, class II, DR α |
| rs3129886 | 32518553 | class II | HLA-DRA | MHC, class II, DR α |
| rs3135391 | 32518964 | class II | HLA-DRA | MHC, class II, DR α |
| rs2239805 | 32519353 | class II | HLA-DRA | MHC, class II, DR α |
| rs2239804 | 32519500 | class II | HLA-DRA | MHC, class II, DR α |
| rs3129888 | 32519703 | class II | HLA-DRA | MHC, class II, DR α |
| rs3135388 | 32521028 | class II |  |  |
| rs2395182 | 32521294 | class II |  |  |
| rs2227139 | 32521436 | class II |  |  |
| rs13209234 | 32523952 | class II | HLA-DRB9 | MHC, class II, DR β 9 (pseudogene) |
| rs6903608 | 32536262 | class II | HLA-DRB9 | MHC, class II, DR β 9 (pseudogene) |
| rs4434496 | 32538485 | class II |  |  |
| rs7766843 | 32538706 | class II |  |  |
| rs2395185 | 32541144 | class II |  |  |
| rs660895 | 32685357 | class II | HLA-DRB1 | MHC, class II, DR β 1 |
| rs532098 | 32686029 | class II |  |  |
| rs3129763 | 32698902 | class II |  |  |
| rs2187668 | 32713861 | class II | HLA-DQA1 | MHC, class II, DQ α 1 |
| rs7744001 | 32734063 | class II |  |  |
| rs9275141 | 32759094 | class II |  |  |
| rs4947342 | 32761047 | class II |  |  |
| rs4642516 | 32765520 | class II |  |  |
| rs6457617 | 32771828 | class II |  |  |
| rs3135006 | 32775096 | class II |  |  |
| rs1794265 | 32782714 | class II |  |  |
| rs2858333 | 32789062 | class II |  |  |
| rs7454108 | 32789460 | class II |  |  |
| rs3916766 | 32789622 | class II |  |  |
| rs3998159 | 32789996 | class II |  |  |
| rs6936863 | 32792006 | class II |  |  |
| rs3916765 | 32793527 | class II |  |  |
| rs2395246 | 32799752 | class II |  |  |
| rs5029394 | 32803059 | class II |  |  |
| rs2858880 | 32812861 | class II |  |  |
| rs7773149 | 32814019 | class II | HLA-DQA2 | MHC, class II, DQ α 2 |
| rs7773955 | 32814696 | class II | HLA-DQA2 | MHC, class II, DQ α 2 |
| rs2213567 | 32819632 | class II | HLA-DQA2 | MHC, class II, DQ α 2 |
| rs9276431 | 32820224 | class II | HLA-DQA2 | MHC, class II, DQ α 2 |
| rs2239800 | 32821244 | class II | HLA-DQA2 | MHC, class II, DQ α 2 |
| rs2395253 | 32823137 | class II | HLA-DQA2 | MHC, class II, DQ α 2 |
| rs4415152 | 32825001 | class II |  |  |
| rs6918223 | 32830509 | class II | HLA-DQB2 | MHC, class II, DQ β 2 |
| rs762815 | 32837619 | class II | HLA-DQB2 | MHC, class II, DQ β 2 |
| rs2071550 | 32838917 | class II | HLA-DQB2 | MHC, class II, DQ β 2 |
| rs1573649 | 32839235 | class II | HLA-DQB2 | MHC, class II, DQ β 2 |
| rs6903130 | 32840187 | class II | HLA-DQB2 | MHC, class II, DQ β 2 |
| rs9276586 | 32840914 | class II |  |  |
| rs9296044 | 32844121 | class II |  |  |
| rs2621421 | 32848648 | class II |  |  |
| rs2621416 | 32849845 | class II |  |  |
| rs2621413 | 32850508 | class II |  |  |
| rs2857177 | 32860817 | class II |  |  |
| rs7758736 | 32866371 | class II |  |  |
| rs2621384 | 32867250 | class II |  |  |
| rs2857150 | 32871798 | class II |  |  |
| rs6899857 | 32878459 | class II |  |  |
| rs2071473 | 32890582 | class II | HLA-DOB | MHC, class II, DO β |
| rs2621326 | 32891873 | class II | HLA-DOB | MHC, class II, DO β |
| rs2857107 | 32893492 | class II |  |  |
| rs1894408 | 32894810 | class II |  |  |
| rs2621323 | 32896684 | class II |  |  |
| rs2856993 | 32899380 | class II | TAP2 | transporter 2, ATP-binding cassette, sub-family B (MDR/TAP) |
| rs1894411 | 32900950 | class II | TAP2 | transporter 2, ATP-binding cassette, sub-family B (MDR/TAP) |
| rs6905503 | 32902096 | class II | TAP2 | transporter 2, ATP-binding cassette, sub-family B (MDR/TAP) |
| rs2857101 | 32902653 | class II | TAP2 | transporter 2, ATP-binding cassette, sub-family B (MDR/TAP) |
| rs10484565 | 32903009 | class II | TAP2 | transporter 2, ATP-binding cassette, sub-family B (MDR/TAP) |
| rs241454 | 32904121 | class II | TAP2 | transporter 2, ATP-binding cassette, sub-family B (MDR/TAP) |
| rs241448 | 32904662 | class II | TAP2 | transporter 2, ATP-binding cassette, sub-family B (MDR/TAP) |
| rs4148876 | 32904770 | class II | TAP2 | transporter 2, ATP-binding cassette, sub-family B (MDR/TAP) |
| rs241438 | 32905597 | class II | TAP2 | transporter 2, ATP-binding cassette, sub-family B (MDR/TAP) |
| rs4576294 | 32906525 | class II | TAP2 | transporter 2, ATP-binding cassette, sub-family B (MDR/TAP) |
| rs241433 | 32906772 | class II | TAP2 | transporter 2, ATP-binding cassette, sub-family B (MDR/TAP) |
| rs9469282 | 32908732 | class II | TAP2 | transporter 2, ATP-binding cassette, sub-family B (MDR/TAP) |
| rs4148871 | 32911293 | class II | TAP2 | transporter 2, ATP-binding cassette, sub-family B (MDR/TAP) |
| rs241429 | 32911817 | class II | TAP2 | transporter 2, ATP-binding cassette, sub-family B (MDR/TAP) |
| rs241424 | 32912911 | class II | TAP2 | transporter 2, ATP-binding cassette, sub-family B (MDR/TAP) |
| rs2071552 | 32914438 | class II | TAP2 | transporter 2, ATP-binding cassette, sub-family B (MDR/TAP) |
| rs3763365 | 32915430 | class II |  |  |
| rs6924102 | 32919360 | class II | PSMB8 | proteasome (prosome, macropain) subunit, β type, 8 (large multifunctional peptidase 7) |
| rs2071540 | 32920893 | class II |  |  |
| rs2071538 | 32926655 | class II | TAP1 | transporter 1, ATP-binding cassette, sub-family B (MDR/TAP) |
| rs2071537 | 32926968 | class II | TAP1 | transporter 1, ATP-binding cassette, sub-family B (MDR/TAP) |
| rs2071481 | 32927842 | class II | TAP1 | transporter 1, ATP-binding cassette, sub-family B (MDR/TAP) |
| rs2071480 | 32929836 | class II |  |  |
| rs4713600 | 32930835 | class II | PSMB9 | proteasome (prosome, macropain) subunit, β type, 9 (large multifunctional peptidase 2) |
| rs3763347 | 32930952 | class II | PSMB9 | proteasome (prosome, macropain) subunit, β type, 9 (large multifunctional peptidase 2) |
| rs2071476 | 32933356 | class II | PSMB9 | proteasome (prosome, macropain) subunit, β type, 9 (large multifunctional peptidase 2) |
| rs7767288 | 32937990 | class II |  |  |
| rs1383266 | 32942709 | class II |  |  |
| rs12214934 | 32944477 | class II |  |  |
| rs7757767 | 32953850 | class II | PPP1R2P1 | protein phosphatase 1, regulatory (inhibitor) subunit 2 pseudogene 1 |
| rs2127675 | 32958827 | class II | PPP1R2P1 | protein phosphatase 1, regulatory (inhibitor) subunit 2 pseudogene 1 |
| rs4947259 | 32959484 | class II |  |  |
| rs2018501 | 32964185 | class II |  |  |
| rs1029295 | 32964459 | class II |  |  |
| rs9469300 | 32968729 | class II |  |  |
| rs241410 | 32969628 | class II |  |  |
| rs3749981 | 32970659 | class II |  |  |
| rs241405 | 32973775 | class II |  |  |
| rs9501234 | 32984026 | class II |  |  |
| rs154989 | 32985150 | class II |  |  |
| rs7769282 | 32986252 | class II |  |  |
| rs154986 | 32987210 | class II |  |  |
| rs241458 | 32996543 | class II |  |  |
| rs154978 | 33007273 | class II |  |  |
| rs154977 | 33007995 | class II |  |  |
| rs181997 | 33008695 | class II |  |  |
| rs3129299 | 33008764 | class II |  |  |
| rs154971 | 33009632 | class II | HLA-DMB | MHC, class II, DM β |
| rs3132132 | 33009911 | class II | HLA-DMB | MHC, class II, DM β |
| rs10751 | 33010560 | class II | HLA-DMB | MHC, class II, DM β |
| rs151719 | 33011877 | class II | HLA-DMB | MHC, class II, DM β |
| rs1042337 | 33012958 | class II | HLA-DMB | MHC, class II, DM β |
| rs714289 | 33013788 | class II | HLA-DMB | MHC, class II, DM β |
| rs580962 | 33033669 | class II |  |  |
| rs10484568 | 33043728 | class II |  |  |
| rs683208 | 33045878 | class II | BRD2 | bromodomain containing 2 |
| rs485502 | 33051322 | class II | BRD2 | bromodomain containing 2 |
| rs3097644 | 33053507 | class II | BRD2 | bromodomain containing 2 |
| rs206781 | 33054110 | class II | BRD2 | bromodomain containing 2 |
| rs2066744 | 33056310 | class II | BRD2 | bromodomain containing 2 |
| rs2071876 | 33056403 | class II | BRD2 | bromodomain containing 2 |
| rs1049526 | 33056781 | class II | BRD2 | bromodomain containing 2 |
| rs188245 | 33063953 | class II |  |  |
| rs206765 | 33072673 | class II |  |  |
| rs172274 | 33077434 | class II |  |  |
| rs1044429 | 33080619 | class II | HLA-DOA | MHC, class II, DO α |
| rs376892 | 33080864 | class II | HLA-DOA | MHC, class II, DO α |
| rs3129304 | 33081720 | class II | HLA-DOA | MHC, class II, DO α |
| rs2582 | 33082528 | class II | HLA-DOA | MHC, class II, DO α |
| rs399604 | 33082991 | class II | HLA-DOA | MHC, class II, DO α |
| rs2284191 | 33084631 | class II | HLA-DOA | MHC, class II, DO α |
| rs86567 | 33084736 | class II | HLA-DOA | MHC, class II, DO α |
| rs6911639 | 33086155 | class II |  |  |
| rs429916 | 33086564 | class II |  |  |
| rs4711313 | 33087723 | class II |  |  |
| rs441126 | 33088555 | class II |  |  |
| rs6457699 | 33089624 | class II |  |  |
| rs413887 | 33090275 | class II |  |  |
| rs2116264 | 33092765 | class II |  |  |
| rs430188 | 33095519 | class II |  |  |
| rs423639 | 33095751 | class II |  |  |
| rs3129301 | 33098037 | class II |  |  |
| rs6920606 | 33105651 | class II |  |  |
| rs3130171 | 33106706 | class II |  |  |
| rs3135195 | 33107307 | class II |  |  |
| rs439852 | 33113185 | class II |  |  |
| rs375912 | 33124705 | class II |  |  |
| rs3130176 | 33125434 | class II |  |  |
| rs588997 | 33136694 | class II | HLA-DPA1 | MHC, class II, DP α 1 |
| rs3077 | 33140999 | class II | HLA-DPA1 | MHC, class II, DP α 1 |
| rs2301226 | 33142573 | class II | HLA-DPA1 | MHC, class II, DP α 1 |
| rs9277341 | 33147602 | class II | HLA-DPA1 | MHC, class II, DP α 1 |
| rs9380340 | 33150268 | class II |  |  |
| rs2071349 | 33151497 | class II |  |  |
| rs3135021 | 33153535 | class II | HLA-DPB1 | MHC, class II, DP β 1 |
| rs3097671 | 33155589 | class II | RPL32P1 | ribosomal protein L32 pseudogene 1 |
| rs9277378 | 33158256 | class II | HLA-DPB1 | MHC, class II, DP β 1 |
| rs3117226 | 33165636 | class II |  |  |
| rs2068204 | 33166695 | class II | LOC646702 | similar to HLA class II histocompatibility antigen, DP α chain precursor (HLA-SB α chain) |
| rs2395314 | 33170650 | class II | LOC646702 | similar to HLA class II histocompatibility antigen, DP α chain precursor (HLA-SB α chain) |
| rs3117213 | 33172582 | class II | LOC646702 | similar to HLA class II histocompatibility antigen, DP α chain precursor (HLA-SB α chain) |
| rs2064476 | 33181299 | class II | LOC646702 | similar to HLA class II histocompatibility antigen, DP α chain precursor (HLA-SB α chain) |
| rs3117234 | 33181961 | class II |  |  |
| rs3128930 | 33183643 | class II |  |  |
| rs6457713 | 33185753 | class II |  |  |
| rs9380343 | 33187143 | class II |  |  |
| rs6937061 | 33187789 | class II |  |  |
| rs2016780 | 33190448 | class II | HLA-DPB2 | MHC, class II, DP β2 (pseudogene) |
| rs3117016 | 33203493 | class II | HLA-DPB2 | MHC, class II, DP β2 (pseudogene) |
| rs3117004 | 33204743 | class II | HLA-DPB2 | MHC, class II, DP β2 (pseudogene) |
| rs3116998 | 33205208 | class II |  |  |
| rs3129270 | 33205400 | class II |  |  |
| rs3129267 | 33206873 | class II |  |  |
| rs2294472 | 33207187 | class II |  |  |
| rs3116985 | 33208567 | class II |  |  |
| rs4711319 | 33215439 | class II |  |  |
| rs7750683 | 33218811 | class II |  |  |
| rs721844 | 33231509 | class II |  |  |
| rs3129207 | 33233289 | Ext class II | COL11A2 | collagen, type XI, α 2 |
| rs3129203 | 33235734 | Ext class II |  |  |
| rs9368757 | 33236354 | Ext class II | COL11A2 | collagen, type XI, α 2 |
| rs2257126 | 33239711 | Ext class II | COL11A2 | collagen, type XI, α 2 |
| rs986522 | 33243939 | Ext class II | COL11A2 | collagen, type XI, α 2 |
| rs986521 | 33244122 | Ext class II | COL11A2 | collagen, type XI, α 2 |
| rs2855442 | 33245380 | Ext class II | COL11A2 | collagen, type XI, α 2 |
| rs9368758 | 33245998 | Ext class II | COL11A2 | collagen, type XI, α 2 |
| rs2072915 | 33270059 | Ext class II | RXRB | retinoid X receptor, β |
| rs464921 | 33348483 | Ext class II | RPS18 | ribosomal protein S18 |
| rs12664430 | 33380654 | Ext class II | TAPBP | TAP binding protein (tapasin) |
