# Supplementary material for: A Major Histocompatibility Class I Locus Contributes to Multiple Sclerosis Susceptibility Independently from HLA-DRB1*15:01
Source: PLoS One. 2010 Jun 25;5(6):e11296. doi: 10.1371/journal.pone.0011296 (PMC2892470; doi:10.1371/journal.pone.0011296)
Supplement: Table S3 — 52 SNPs significantly associated with MS susceptibility in the discovery and replication datasets using Cochran Armitage trend test, FDR = .05, adjusted for sex, center (US versus UK) and HLA-DRB1*15:01. The SNPs are listed in order of chromosomal position from telomere to centromere. The p-values for the merged dataset are unadjusted. rs2523393 is a tagging SNP for HLA-B*44:02 [12], [14]. (0.17 MB DOC) [file pone.0011296.s004.doc]

| SNP Name | Position | SNP | Gene | Discovery | Replication | Merged | Major | Cases | Controls |
| --- | --- | --- | --- | --- | --- | --- | --- | --- | --- |
|  |  |  |  | P Value | P Value | P Value | Allele | MAF | MAF |
| Class I SNP Associations | | | | | | | | | |
| rs2256266 | 29740296 | A/G | MOG | 0.00349 | 1.41 X 10^-6^ | 3.68 X 10^-8^ | G | 0.18 | 0.22 |
| rs2747457 | 29764395 | A/C |  | 0.00120 | 0.00952 | 0.00004 | A | 0.25 | 0.23 |
| rs3131865 | 29780143 | C/G |  | 0.00060 | 0.01566 | 0.00004 | G | 0.29 | 0.27 |
| rs3094724 | 29782273 | A/G |  | 0.00360 | 0.01448 | 0.00016 | A | 0.23 | 0.21 |
| rs1362126 | 29798997 | A/G | HLA-F | 0.00002 | 0.00023 | 1.48 X 10^-8^ | G | 0.35 | 0.41 |
| rs2523393* | 29813637 | C/T | FLJ35429 | 0.00003 | 1.97 X 10^-5^ | 2.07 X 10^-9^ | T | 0.38 | 0.44 |
| rs2743951 | 29817212 | C/T | FLJ35429 | 0.00005 | 1.89 X 10^-5^ | 3.08 X 10^-9^ | C | 0.38 | 0.44 |
| rs1736936 | 29902295 | C/T | HCG4P8 | 0.00045 | 1.71 X 10^-5^ | 2.39 X 10^-8^ | C | 0.50 | 0.47 |
| rs2523822 | 29936638 | A/G |  | 0.00023 | 1.81 X 10^-7^ | 2.08 X 10^-10^ | A | 0.23 | 0.28 |
| rs1611710 | 29936894 | C/T |  | 0.00030 | 0.00033 | 2.06 X 10^-7^ | C | 0.46 | 0.44 |
| rs2734971 | 29942427 | C/T | 3.8-1.4 | 0.00188 | 1.31 X 10^-5^ | 7.81 X 10^-8^ | C | 0.44 | 0.46 |
| rs2517701 | 30033950 | A/T | HLA-80 | 0.00008 | 3.85 X 10^-5^ | 1.74 X 10*^-8^* | A | 0.26 | 0.32 |
| rs4713270 | 30042675 | A/G | HCG2P6 | 0.00012 | 6.53 X 10^-7^ | 3.68 X 10^-10^ | G | 0.23 | 0.28 |
| rs4713274 | 30045471 | C/G | MICD | 0.00005 | 4.63 X 10^-13^ | 8.20 X 10^-12^ | C | 0.21 | 0.27 |
| rs2256543 | 30045811 | A/G | MICD | 4.95 X 10^-6^ | 0.00024 | 7.85 X 10^-9^ | G | 0.50 | 0.43 |
| rs2523946 | 30049921 | C/T | MICD | 0.00006 | 0.00041 | 8.76 X 10^-8^ | C | 0.39 | 0.45 |
| rs3823355 | 30050061 | C/T | MICD | 0.00021 | 5.43 X 10^-7^ | 5.82 X 10^-10^ | C | 0.22 | 0.28 |
| rs4959039 | 30065047 | A/G | HLA-G | 0.00004 | 4.03 X 10^-8^ | 8.65 X 10^-12^ | A | 0.21 | 0.26 |
| rs4713281 | 30086330 | A/G | HLA-J | 0.00005 | 1.04 X 10^-9^ | 7.76 X 10^-12^ | G | 0.21 | 0.27 |
| rs9357092 | 30092230 | A/G | HCG9 | 0.00004 | 7.37 X 10^-8^ | 1.65 X 10^-11^ | G | 0.21 | 0.27 |
| rs259943 | 30123309 | A/G |  | 0.00081 | 0.00011 | 0.00032 | A | 0.30 | 0.28 |
| rs9393989 | 30148062 | A/G | RNF39 | 0.00005 | 3.65 X 10^-8^ | 1.19 X 10^-11^ | G | 0.21 | 0.27 |
| rs2523990 | 30185207 | A/G | TRIM31 | 0.00016 | 0.00067 | 4.75 X 10^-7^ | G | 0.47 | 0.47 |
| rs1541268 | 30211372 | C/T | TRIM40 | 0.00271 | 1.03 x 10^-6^ | 1.87 X 10^-8^ | T | 0.18 | 0.23 |
| rs1557608 | 30226560 | G/T | TRIM40 | 0.00296 | 0.00068 | 7.16 X 10^-6^ | T | 0.23 | 0.28 |
| rs1029239 | 30246140 | C/G | TRIM15 | 0.00300 | 0.00642 | 0.00005 | G | 0.47 | 0.46 |
| rs2239530 | 30260093 | C/G | TRIM26 | 0.00133 | 4.97 X 10^-7^ | 4.44 X 10^-9^ | G | 0.18 | 0.22 |
| rs2844775 | 30287400 | A/G | TRIM26 | 0.00363 | 3.40 X 10^-5^ | 4.89 X 10^-7^ | G | 0.20 | 0.24 |
| rs2394390 | 30709799 | A/G | PTMAP1 | 0.00198 | 0.00962 | 0.00109 | A | 0.04 | 0.04 |
| rs4713433 | 31176005 | A/C |  | 0.00092 | 9.12 X 10^-5^ | 0.00022 | C | 0.14 | 0.18 |
| rs2394885 | 31282569 | C/G |  | 0.00004 | 1.35 X 10^-5^ | 8.09 X 10^-7^ | C | 0.11 | 0.16 |
| Class III SNP Associations | | | | | | | | | |
| rs2471980 | 31908846 | C/G | HSPA1B | 0.04310 | 8.51 X 10^-6^ | 3.80 X 10^-6^ | G | 0.30 | 0.31 |
| rs660550 | 31945255 | G/T | SLC44A4 | 0.01912 | 0.01354 | 0.00065 | T | 0.47 | 0.42 |
| rs3130481 | 31947734 | C/G | SLC44A4 | 0.04791 | 1.17 X 10^-5^ | 0.00101 | C | 0.45 | 0.45 |
| rs2763982 | 31980529 | C/G |  | 0.03594 | 0.00008 | 0.00002 | G | 0.27 | 0.36 |
| rs2071285 | 32288408 | A/T | NOTCH4 | 0.00298 | 0.00124 | 0.00002 | A | 0.04 | 0.07 |
| rs206015 | 32290736 | A/G | NOTCH4 | 0.00402 | 0.00618 | 0.00006 | G | 0.08 | 0.12 |
| rs384247 | 32292551 | A/G | NOTCH4 | 0.00316 | 0.00363 | 0.00003 | G | 0.12 | 0.17 |
| Class II SNP Associations | | | | | | | | | |
| rs9268148 | 32367504 | A/G | TSBP | 0.00023 | 1.68 X 10^-6^ | 6.21 X 10^-10^ | A | 0.35 | 0.17 |
| rs3132958 | 32405878 | A/G | TSBP | 0.00774 | 0.00102 | 0.00002 | G | 0.40 | 0.24 |
| rs3129904 | 32418373 | A/G | TSBP | 0.00865 | 0.00098 | 0.00003 | G | 0.40 | 0.24 |
| rs3132963 | 32428130 | A/G | TSBP | 1.37 X 10^-5^ | 8.23 X 10^-8^ | 1.36 X 10^-11^ | A | 0.35 | 0.17 |
| rs2050191 | 32446878 | A/T | TSBP | 0.01391 | 0.00071 | 0.00003 | T | 0.40 | 0.24 |
| rs3129961 | 32486917 | A/G |  | 0.01484 | 1.21 X 10^-5^ | 0.00514 | A | 0.44 | 0.30 |
| rs3135352 | 32500883 | G/T |  | 4.66 X 10^-6^ | 0.01187 | 2.57 X 10^-7^ | T | 0.31 | 0.14 |
| rs3135391 | 32518964 | C/T | HLA-DRA | 2.79 X 10^-6^ | 0.00572 | 7.32 X 10^-8^ | C | 0.31 | 0.14 |
| rs3129888 | 32519703 | A/G | HLA-DRA | 0.00013 | 0.00180 | 6.77 X 10^-7^ | A | 0.37 | 0.20 |
| rs3135388 | 32521028 | A/G |  | 1.79 X 10^-6^ | 0.00612 | 5.50 X 10^-8^ | G | 0.31 | 0.14 |
| rs2395182 | 32521294 | G/T |  | 0.00006 | 0.00125 | 2.59 X 10^-7^ | T | 0.39 | 0.22 |
| rs2227139 | 32521436 | A/G |  | 0.00087 | 0.00006 | 2.60 X 10^-7^ | A | 0.46 | 0.39 |
| rs2071876 | 33056403 | C/T | BRD2 | 0.00232 | 2.44 X 10^-6^ | 5.70 X 10^-8^ | C | 0.06 | 0.10 |
| rs4711319 | 33215439 | A/G |  | 6.15 X 10^-6^ | 0.00033 | 5.02 X 10^-6^ | G | 0.12 | 0.17 |
