# Supplementary material for: A Major Histocompatibility Class I Locus Contributes to Multiple Sclerosis Susceptibility Independently from HLA-DRB1*15:01
Source: PLoS One. 2010 Jun 25;5(6):e11296. doi: 10.1371/journal.pone.0011296 (PMC2892470; doi:10.1371/journal.pone.0011296)
Supplement: Table S4 — 48 SNPs significantly associated with MS susceptibility in the merged HLA-DRB1*15:01 (-) dataset, using the trend test and adjusting for sex, center (US versus UK) and dataset (discovery versus replication). SNPs are listed in order of most to least statistical significance. Four class II SNPs identified in the discovery and replication datasets were no longer significantly associated with MS susceptibility in the HLA-DRB1*15:01 (-) dataset: rs3129961, rs3135352, rs3135391, and rs3135388. (0.14 MB DOC) [file pone.0011296.s005.doc]

| Merged Cohort *DRB1*15:01*(-) Subjects | | | | | | | | |
| --- | --- | --- | --- | --- | --- | --- | --- | --- |
|  |  |  |  | MS Associated | Trend | Odds | 95% CI | |
| SNP Name | Position | Class | Gene | Allele | P Values | Ratio | Lower | Upper |
| rs4959039 | 30065047 | class I | HLA-G | A | 8.45 X 10^-13^ | 1.59 | 1.40 | 1.81 |
| rs9393989 | 30148062 | class I | RNF39 | A | 9.84 X 10^-13^ | 0.63 | 0.56 | 0.72 |
| rs9357092 | 30092230 | class I | HCG9 | A | 1.17 X 10^-12^ | 0.63 | 0.56 | 0.72 |
| rs4713281 | 30086330 | class I | HLA-J | A | 3.19 X 10^-12^ | 0.63 | 0.56 | 0.72 |
| rs4713274 | 30045471 | class I | MICD | C | 5.11 X 10^-12^ | 1.56 | 1.38 | 1.77 |
| rs1736936 | 29902295 | class I | HCG4P8 | C | 2.22 X 10^-11^ | 0.70 | 0.63 | 0.78 |
| rs2523822 | 29936638 | class I |  | A | 2.99 X 10^-11^ | 1.51 | 1.34 | 1.70 |
| rs4713270 | 30042675 | class I | HCG2P6 | A | 4.26 X 10^-11^ | 0.66 | 0.58 | 0.75 |
| rs3823355 | 30050061 | class I | MICD | C | 7.74 X 10^-11^ | 1.50 | 1.33 | 1.70 |
| rs2734971 | 29942427 | class I | 3.8-1.4 | C | 2.07 X 10^-10^ | 1.42 | 1.27 | 1.58 |
| rs2239530 | 30260093 | class I | TRIM26 | C | 2.96 X 10^-10^ | 0.65 | 0.57 | 0.74 |
| rs2523393 | 29813637 | class I | FLJ35429 | C | 6.04 X 10^-10^ | 0.72 | 0.65 | 0.80 |
| rs1541268 | 30211372 | class I | TRIM40 | C | 1.40 X 10^-9^ | 0.66 | 0.58 | 0.76 |
| rs2256266 | 29740296 | Ext Cls I | MOG | A | 2.67 X 10^-9^ | 0.66 | 0.58 | 0.76 |
| rs2743951 | 29817212 | class I | FLJ35429 | C | 3.55 X 10^-9^ | 1.37 | 1.24 | 1.52 |
| rs1611710 | 29936894 | class I |  | C | 5.06 X 10^-9^ | 0.74 | 0.66 | 0.82 |
| rs2517701 | 30033950 | class I | HLA-80 | A | 5.62 X 10^-9^ | 1.41 | 1.26 | 1.58 |
| rs2523946 | 30049921 | class I | MICD | C | 8.69 X 10^-9^ | 1.36 | 1.23 | 1.51 |
| rs2256543 | 30045811 | class I | MICD | A | 9.15 X 10^-9^ | 1.36 | 1.22 | 1.51 |
| rs1362126 | 29798997 | class I | HLA-F | A | 6.99 X 10^-9^ | 0.75 | 0.67 | 0.83 |
| rs1557608 | 30226560 | class I | TRIM40 | G | 1.51 X 10^-7^ | 0.72 | 0.64 | 0.82 |
| rs2844775 | 30287400 | class I | TRIM26 | A | 2.47 X 10^-7^ | 0.71 | 0.63 | 0.81 |
| rs9268148 | 32367504 | class II | TSBP | A | 9.68 X 10^-7^ | 0.58 | 0.47 | 0.72 |
| rs1029239 | 30246140 | class I | TRIM15 | C | 1.48 X 10^-6^ | 1.29 | 1.17 | 1.44 |
| rs2523990 | 30185207 | class I | TRIM31 | A | 1.56 X 10^-6^ | 1.29 | 1.16 | 1.43 |
| rs3132963 | 32428130 | class II | TSBP | A | 3.73 X 10^-6^ | 0.58 | 0.46 | 0.73 |
| rs2747457 | 29764395 | class I |  | A | 1.11 X 10^-5^ | 0.77 | 0.69 | 0.87 |
| rs2394885 | 31282569 | class I |  | C | 3.27 X 10^-5^ | 1.37 | 1.18 | 1.59 |
| rs2471980 | 31908846 | class III | HSPA1B | C | 3.71 X 10^-5^ | 1.25 | 1.12 | 1.39 |
| rs3094724 | 29782273 | class I |  | A | 5.08 X 10^-5^ | 0.78 | 0.69 | 0.88 |
| rs2227139 | 32521436 | class II |  | A | 6.09 X 10^-5^ | 0.80 | 0.71 | 0.89 |
| rs259943 | 30123309 | class I |  | A | 6.09 X 10^-5^ | 0.80 | 0.71 | 0.89 |
| rs3131865 | 29780143 | class I |  | C | 0.000104 | 1.25 | 1.12 | 1.40 |
| rs2071876 | 33056403 | class II | BRD2 | C | 0.000130 | 1.44 | 1.20 | 1.74 |
| rs4711319 | 33215439 | class II |  | A | 0.000241 | 0.77 | 0.66 | 0.88 |
| rs2071285 | 32288408 | class III | NOTCH4 | A | 0.000347 | 1.48 | 1.19 | 1.83 |
| rs206015 | 32290736 | class III | NOTCH4 | A | 0.001612 | 0.78 | 0.66 | 0.91 |
| rs4713433 | 31176005 | class I |  | A | 0.001837 | 0.80 | 0.70 | 0.92 |
| rs2763982 | 31980529 | class III |  | C | 0.002970 | 0.85 | 0.77 | 0.95 |
| rs2395182 | 32521294 | class II |  | G | 0.003431 | 1.29 | 1.09 | 1.52 |
| rs384247 | 32292551 | class III | NOTCH4 | A | 0.005644 | 0.83 | 0.72 | 0.95 |
| rs2394390 | 30709799 | class I | PTMAP1 | A | 0.00778 | 0.73 | 0.58 | 0.92 |
| rs3132958 | 32405878 | class II | TSBP | A | 0.02631 | 1.18 | 1.02 | 1.37 |
| rs660550 | 31945255 | class III | SLC44A4 | G | 0.03033 | 1.13 | 1.01 | 1.25 |
| rs3129904 | 32418373 | class II | TSBP | A | 0.03070 | 1.18 | 1.02 | 1.36 |
| rs3130481 | 31947734 | class III | SLC44A4 | C | 0.03805 | 0.90 | 0.81 | 0.99 |
| rs3129888 | 32519703 | class II | HLA-DRA | A | 0.04732 | 0.82 | 0.68 | 1.00 |
| rs2050191 | 32446878 | class II | TSBP | A | 0.04745 | 1.16 | 1.00 | 1.34 |
