# Supplementary material for: A Major Histocompatibility Class I Locus Contributes to Multiple Sclerosis Susceptibility Independently from HLA-DRB1*15:01
Source: PLoS One. 2010 Jun 25;5(6):e11296. doi: 10.1371/journal.pone.0011296 (PMC2892470; doi:10.1371/journal.pone.0011296)
Supplement: Table S5 — 48 SNPs that are associated with MS susceptibility in the HLA-DRB1*15:01(-) dataset are grouped together using an algorithm to define SNP clusters based on LD-R2≥.05 (moderate to strong LD) [15]. The 48 SNPs can be grouped into 20 SNP clusters and tagging SNPs for each cluster are designated by an asterisk. The SNPs are listed in order of cluster size with the largest cluster including 10 SNPs and the smallest SNP clusters include only single SNPs. (0.09 MB DOC) [file pone.0011296.s006.doc]

| **SNP Clusters LD-R^2^≤.05** | | | |
| --- | --- | --- | --- |
| **SNP Name** | **Position** | **Class** | **Gene** |
| rs2256266 | 29740296 | Ext Cls I | MOG |
| rs2523822 | 29936638 | class I |  |
| rs2517701 | 30033950 | class I | HLA-80 |
| rs4713270 | 30042675 | class I | HCG2P6 |
| rs4713274 | 30045471 | class I | MICD |
| rs3823355 | 30050061 | class I | MICD |
| rs4959039* | 30065047 | class I | HLA-G |
| rs4713281 | 30086330 | class I | HLA-J |
| rs9357092 | 30092230 | class I | HCG9 |
| rs9393989 | 30148062 | class I | RNF39 |
| rs2747457 | 29764395 | class I |  |
| rs3131865 | 29780143 | class I |  |
| rs3094724* | 29782273 | class I |  |
| rs259943 | 30123309 | class I |  |
| rs1541268 | 30211372 | class I | TRIM40 |
| rs1557608 | 30226560 | class I | TRIM40 |
| rs2239530* | 30260093 | class I | TRIM26 |
| rs2844775 | 30287400 | class I | TRIM26 |
| rs1362126 | 29798997 | class I | HLA-F |
| rs2523393* | 29813637 | class I | FLJ35429 |
| rs2743951 | 29817212 | class I | FLJ35429 |
| rs1736936 | 29902295 | class I | HCG4P8 |
| rs1611710* | 29936894 | class I |  |
| rs2734971 | 29942427 | class I | 3.8-1.4 |
| rs2071285 | 32288408 | class III | NOTCH4 |
| rs206015* | 32290736 | class III | NOTCH4 |
| rs384247 | 32292551 | class III | NOTCH4 |
| rs3132958 | 32405878 | class II | C6orf10 |
| rs3129904* | 32418373 | class II | C6orf10 |
| rs2050191 | 32446878 | class II | C6orf10 |
| rs2256543* | 30045811 | class I | MICD |
| rs2523946 | 30049921 | class I | MICD |
| rs4713433* | 31176005 | class I |  |
| rs2394885 | 31282569 | class I |  |
| rs660550* | 31945255 | class III | SLC44A4 |
| rs3130481 | 31947734 | class III | SLC44A4 |
| rs9268148 | 32367504 | class II | C6orf10 |
| rs3132963* | 32428130 | class II | C6orf10 |
| rs3129888 | 32519703 | class II | HLA-DRA |
| rs2395182* | 32521294 | class II |  |
| rs2523990* | 30185207 | class I | TRIM31 |
| rs1029239* | 30246140 | class I | TRIM15 |
| rs2394390* | 30709799 | class I | PTMAP1 |
| rs2471980* | 31908846 | class III | HSPA1B |
| rs2763982* | 31980529 | class III |  |
| rs2227139* | 32521436 | class II |  |
| rs2071876* | 33056403 | class II | BRD2 |
| rs4711319* | 33215439 | class II |  |
