# Supplementary material for: A Major Histocompatibility Class I Locus Contributes to Multiple Sclerosis Susceptibility Independently from HLA-DRB1*15:01
Source: PLoS One. 2010 Jun 25;5(6):e11296. doi: 10.1371/journal.pone.0011296 (PMC2892470; doi:10.1371/journal.pone.0011296)
Supplement: Table S6 — Two locus haplotypes for the SNPs rs2523393 (tags HLA-B*44:02) and SNP rs459039 (near HLA-G). A MS risk haplotype is rs2523393:T>C with rs4959039:A>G and a MS protective haplotype is rs2523393:C>T with rs4959039:G>A. The heterozygous haplotype is appears to be protective suggesting a dominant effect of the protective haplotype. The p-values and odds ratios are adjusted for the covariates sex (men versus women) and cohort (discovery versus replication) to control for stratification. (0.05 MB DOC) [file pone.0011296.s007.doc]

| **rs2523393** | **rs4959039** |  |  |  |  |  |  |
| --- | --- | --- | --- | --- | --- | --- | --- |
| **(HLA-B*4402)** | **(near HLA-G)** | **Cases (N)** | **Controls (N)** | **% Cases** | **% Controls** | **Odds Ratio** | **P-Value** |
| T/T | A/A | 425 | 687 | 39.28 | 29.11 | 1.58 | 2.97 X 10^-9^ |
| T/T | A/G | 17 | 40 | 1.57 | 1.69 | 0.91 | 0.7413 |
| T/T | G/G | 0 | 1 | 0.00 | 0.04 |  |  |
| C/T | A/A | 260 | 497 | 24.03 | 21.06 | 1.18 | 0.0627 |
| C/T | A/G | 218 | 634 | 20.15 | 26.86 | 0.71 | 9.73 X 10^-5^ |
| C/T | G/G | 7 | 26 | 0.65 | 1.10 | 0.60 | 0.2384 |
| C/C | A/A | 40 | 90 | 3.70 | 3.81 | 0.98 | 0.9051 |
| C/C | A/G | 78 | 240 | 7.21 | 10.17 | 0.65 | 0.0018 |
| C/C | G/G | 37 | 145 | 3.42 | 6.14 | 0.52 | 0.0006 |
