# Supplementary material for: A Major Histocompatibility Class I Locus Contributes to Multiple Sclerosis Susceptibility Independently from HLA-DRB1*15:01
Source: PLoS One. 2010 Jun 25;5(6):e11296. doi: 10.1371/journal.pone.0011296 (PMC2892470; doi:10.1371/journal.pone.0011296)
Supplement: Table S7 — SNPs significantly associated with MS susceptibility in the HLA-G locus typed in an independent dataset used for a genome wide meta-analysis. (0.12 MB DOC) [file pone.0011296.s008.doc]

| **SNP Name** | **Position** | **Alleles** | ***p*-Value** | **Location** |
| --- | --- | --- | --- | --- |
| rs1611715 | 29937461 | C,A | 8.57 X 10^-9^ | Centromeric |
| rs3115627 | 29928257 | G,A | 2.93 X 10^-8^ | Centromeric |
| rs2734982 | 29929546 | T,G | 6.28 X 10^-7^ | Centromeric |
| rs2975033 | 29930240 | A,G | 6.37 X 10^-7^ | Centromeric |
| rs1611627 | 29905761 | T,C | 9.73 X 10^-6^ | *HLA-G* intron |
| rs1063320 | 29906728 | C,G | 3.47 X 10^-5^ | *HLA-G* exon |
| rs915668 | 29906438 | C,G | 4.19 X 10^-5^ | *HLA-G* intron |
| rs1736920 | 29905152 | A,G | 4.80 X 10^-5^ | *HLA-G* intron |
| rs1632942 | 29904619 | T,C | 5.39 X 10^-5^ | Centromeric |
| rs2517830 | 29937545 | T,G | 5.97 X 10^-5^ | Centromeric |
| rs1736935 | 29902422 | A,G | 6.09 X 10^-5^ | *HLA-G* intron |
| rs1610678 | 29897169 | T,C | 6.90 X 10^-5^ | Telomeric |
| rs1611149 | 29897978 | C,T | 7.07 X 10^-5^ | Telomeric |
| rs1632933 | 29905912 | C,T | 8.49 X 10^-5^ | *HLA-G* intron |
| rs1611674 | 29933413 | A,G | 8.80 X 10^-5^ | Centromeric |
| rs1736936 | 29902296 | G,A | 0.00010 | Telomeric |
| rs1610677 | 29897150 | T,C | 0.00011 | Telomeric |
| rs2523756 | 29928696 | A,G | 0.00024 | Centromeric |
| rs1611710 | 29936895 | C,T | 0.00024 | Centromeric |
| rs1611705 | 29936472 | T,G | 0.00024 | Centromeric |
| rs1611699 | 29935732 | T,A | 0.00024 | Centromeric |
| rs1611670 | 29933218 | C,T | 0.00025 | Centromeric |
| rs1611666 | 29932824 | T,A | 0.00025 | Centromeric |
| rs9258689 | 29932266 | C,G | 0.00025 | Centromeric |
| rs9258681 | 29932075 | G,T | 0.00025 | Centromeric |
| rs9258679 | 29932038 | A,G | 0.00025 | Centromeric |
| rs6919513 | 29931973 | C,T | 0.00025 | Centromeric |
| rs2517849 | 29931798 | G,A | 0.00025 | Centromeric |
| rs6939251 | 29931708 | G,A | 0.00025 | Centromeric |
| rs2587152 | 29938987 | C,T | 0.00025 | Centromeric |
| rs2734979 | 29931140 | A,G | 0.00026 | Centromeric |
| rs2428510 | 29931006 | G,A | 0.00026 | Centromeric |
| rs9258651 | 29930334 | C,T | 0.00026 | Centromeric |
| rs1611737 | 29939550 | C,G | 0.00026 | Centromeric |
| rs1611739 | 29939777 | T,G | 0.00026 | Centromeric |
| rs1611723 | 29938484 | A,G | 0.00027 | Centromeric |
| rs9404952 | 29912144 | A,G | 0.00037 | Centromeric |
| rs2517851 | 29931761 | C,T | 0.00053 | Centromeric |
| rs1736939 | 29901364 | G,A | 0.00070 | Telomeric |
| rs2735024 | 29900467 | T,C | 0.00071 | Telomeric |
| rs407238 | 29914880 | G,C | 0.00101 | Centromeric |
| rs2734983 | 29928930 | T,A | 0.00143 | Centromeric |
| rs1611714 | 29937386 | G,A | 0.00144 | Centromeric |
| rs1611703 | 29936414 | A,G | 0.00145 | Centromeric |
| rs1611701 | 29935935 | C,T | 0.00146 | Centromeric |
| rs1611689 | 29934705 | T,A | 0.00147 | Centromeric |
| rs1611678 | 29933489 | A,G | 0.00148 | Centromeric |
| rs2508049 | 29931862 | A,G | 0.00149 | Centromeric |
| rs2517866 | 29929011 | C,T | 0.00149 | Centromeric |
| rs9258636 | 29929816 | C,T | 0.00150 | Centromeric |
| rs2517862 | 29929916 | C,T | 0.00151 | Centromeric |
| rs1611738 | 29939685 | C,A | 0.00154 | Centromeric |
| rs2508052 | 29927981 | C,A | 0.00177 | Centromeric |
| rs9258642 | 29930049 | C,T | 0.00219 | Centromeric |
| rs1611709 | 29936865 | T,C | 0.00290 | Centromeric |
| rs2249571 | 29900554 | A,G | 0.00863 | Telomeric |
| rs2735014 | 29913788 | C,A | 0.00885 | Centromeric |
| rs2735011 | 29914985 | T,C | 0.00892 | Centromeric |
| rs2517887 | 29915372 | G,C | 0.00892 | Centromeric |
| rs2735008 | 29915992 | G,A | 0.00893 | Centromeric |
| rs2743937 | 29915902 | T,C | 0.00893 | Centromeric |
| rs2735007 | 29916178 | G,A | 0.00893 | Centromeric |
| rs2735003 | 29916613 | T,G | 0.00893 | Centromeric |
